# Characterizing cognitive aging in humans with links to animal models

**DOI:** 10.3389/fnagi.2012.00021

**Published:** 2012-09-12

**Authors:** Gene E. Alexander, Lee Ryan, Dawn Bowers, Thomas C. Foster, Jennifer L. Bizon, David S. Geldmacher, Elizabeth L. Glisky

**Affiliations:** ^1^Department of Psychology, Evelyn F. McKnight Brain Institute, University of ArizonaTucson, AZ, USA; ^2^Department of Clinical and Health Psychology, University of FloridaGainesville, FL, USA; ^3^Department of Neurology, University of FloridaGainesville, FL, USA; ^4^McKnight Brain Institute, University of FloridaGainesville, FL, USA; ^5^Department of Neuroscience, University of FloridaGainesville, FL, USA; ^6^Departments of Neurology and Neurobiology, Evelyn F. McKnight Brain Institute, University of Alabama at BirminghamBirmingham, AL, USA

**Keywords:** aging, cognition, memory, executive function, processing speed, visuospatial function, language

## Abstract

With the population of older adults expected to grow rapidly over the next two decades, it has become increasingly important to advance research efforts to elucidate the mechanisms associated with cognitive aging, with the ultimate goal of developing effective interventions and prevention therapies. Although there has been a vast research literature on the use of cognitive tests to evaluate the effects of aging and age-related neurodegenerative disease, the need for a set of standardized measures to characterize the cognitive profiles specific to healthy aging has been widely recognized. Here we present a review of selected methods and approaches that have been applied in human research studies to evaluate the effects of aging on cognition, including executive function, memory, processing speed, language, and visuospatial function. The effects of healthy aging on each of these cognitive domains are discussed with examples from cognitive/experimental and clinical/neuropsychological approaches. Further, we consider those measures that have clear conceptual and methodological links to tasks currently in use for non-human animal studies of aging, as well as those that have the potential for translation to animal aging research. Having a complementary set of measures to assess the cognitive profiles of healthy aging across species provides a unique opportunity to enhance research efforts for cross-sectional, longitudinal, and intervention studies of cognitive aging. Taking a cross-species, translational approach will help to advance cognitive aging research, leading to a greater understanding of associated neurobiological mechanisms with the potential for developing effective interventions and prevention therapies for age-related cognitive decline.

## Introduction

Preserving our cognitive abilities is an essential part of maintaining a high quality of life as we age. Although an extensive and varied literature exists in the use of cognitive measures to assess the effects of aging and age-related neurological disease, the development of a standardized and widely available set of tests to characterize the profiles associated with healthy cognitive aging has been recognized as an important direction for advancing future research (Wagster, 2009). It is well established that healthy aging is associated with declines in aspects of memory, executive function, and information processing speed, as well as other selected cognitive abilities (Zec, 1995; Lezak et al., 2012). There are, however, substantial individual differences in the ability of elders to maintain their cognitive functions during aging. This heterogeneity can be viewed as a continuum extending from “successful cognitive aging” with the ability to maintain high levels of functioning throughout the lifespan, to pathological aging, with impairment in memory and other cognitive abilities often leading to dementia (Daffner, 2010). Understanding why some individuals demonstrate successful cognitive aging and others do not may provide an essential foundation for developing effective interventions to enhance the abilities and quality of life for the rapidly growing population of community-dwelling elderly.

The opportunities to relate tests of cognitive abilities to current and emerging measures of neural system integrity and function, to genetic variability and biological risk factors, and to health and lifestyle characteristics may lead to the development of useful biomarkers of cognitive aging. The development of such biomarkers to evaluate the effects of healthy aging on cognition complements recent efforts to identify optimal markers of age-related neurodegenerative disease (e.g., Jack et al., 2008). The availability of biomarkers that are sensitive to the early cognitive effects of aging and that can help track their longitudinal progression will be important for evaluating targeted interventions and prevention therapies designed to delay or diminish declines associated with cognitive aging. Further, using tests that can be conceptually and methodologically linked to measures used in studies of non-human animal models of aging provides a valuable and unique opportunity to advance understanding of the underlying neural mechanisms that influence cognitive aging. In this context, studies of aging in non-human primates and rodents provide an important complement to human studies with the potential for identifying the underlying neurobiological substrates of cognitive aging (see Moss et al., 2007; LaSarge and Nicolle, 2009). This translational approach to cognitive aging research may help to identify new molecular pathways and targets for the development of effective interventions and prevention therapies.

To support and advance such translational research in cognitive aging, we propose the need for the development and implementation of a set of measures to characterize cognitive profiles that are: (1) able to evaluate group differences in both cross-sectional and longitudinal studies of healthy aging; (2) sensitive to individual differences in performance within the continuum of healthy cognitive aging; (3) able to characterize the broad range of abilities across multiple cognitive domains that may be distinct from the impact of both incipient and clinically evident neurological disease; and (4) able to support cross-species translation between human and non-human animal models of aging to advance research into the underlying neural mechanisms of cognitive aging.

In this article, we present a selected review of methods and approaches used to evaluate the higher-order cognitive processes altered by healthy cognitive aging. We focus on five major cognitive domains, including executive function, memory, processing speed, language, and visuospatial function. Well-established age effects are discussed, while also considering aspects of those domains that can remain relatively preserved to provide the potential for developing a characteristic multi-domain profile of cognitive aging. For each domain, perspectives from both cognitive/experimental and clinical/neuropsychological approaches are presented. Further, we consider those measures that have linkages to tasks currently in use in studies of animal models of aging, as well as those that have the promise of being readily translated for research with non-human primates and rodent studies of aging. Our goal is not to provide a comprehensive description of tests for animal models, but rather to suggest human tests for studies of cognitive aging with unique potential for linkages to animal paradigms. Many of these tasks are also described in more detail in the companion papers in this issue. Together with important contributions by the NIH Toolbox for Assessment of Neurological and Behavioral Function (www.nihtoolbox.org) and other related initiatives, such measures may provide an array of complementary methodological tools that will help to greatly advance cognitive aging research, with the ultimate goal of identifying effective interventions and prevention therapies for age-related cognitive decline.

## Executive function

Numerous approaches and models have attempted to characterize executive function in humans. Many suggest that there may be some overarching unitary function that captures what we mean by executive function, such as attentional control (e.g., Kane et al., 2007) or goal maintenance (Braver and West, 2008). Subsumed under this general rubric, however, is a diversity of processes that are engaged differentially depending on task demands. Based on converging evidence from the cognitive/experimental, clinical/neuropsychological, and neuroimaging literatures, we identified three sub-processes of executive function—task-switching, updating, and inhibition—that have been associated with different brain regions in prefrontal cortex and appear to be negatively affected by normal and pathological aging. In this section, we discuss the supporting evidence for the existence of these specific executive processes and propose a set of tests to measure them. We also include working memory, focusing on its executive components.

### Evidence from cognitive/experimental studies

The most influential model of executive function emerging from the cognitive/experimental approach is a model proposed by Miyake et al. (2000) based on confirmatory factor analysis (CFA) and structural equation modeling (SEM). Miyake et al.'s model, based on 137 young adults, identified three latent factors that they called task or set shifting, updating and monitoring of working memory, and inhibition of prepotent responses, with three tasks loading on each factor. The model suggests that these three latent factors tap separable components of executive function, but they are also correlated with each other suggesting they may share common variance as well. A subsequent SEM looked at the extent to which these latent variables predicted performance on more complex executive function tasks. This analysis indicated that shifting uniquely predicted perseverative errors on the Wisconsin Card Sorting Test, updating predicted performance on Operation Span, and inhibition predicted performance on the Tower of Hanoi task. Both updating and inhibition predicted performance on Random Number Generation (RNG).

Fisk and Sharp (2004), in a subsequent principal components analysis of a subset of the complex executive function tasks (WCST, span tasks, and RNG) in a group of 95 adults aged 20–81, obtained a similar factor structure across all ages, plus a fourth factor, which they called efficiency of lexical access (measured by verbal fluency). Vaughan and Giovanello (2010) in a study of 95 older adults, aged 60–90 years old, confirmed the same three-factor structure as obtained by Miyake et al. (see also Latzman and Markon, 2010) and further showed that the executive function latent variables, particularly task switching, predicted performance on instrumental activities of daily living (IADLs; see also Bell-McGinty et al., 2002). Two other studies with older adults found two-factor structures: Hedden and Yoon (2006) found a combined shifting/updating factor and a separate inhibition of proactive interference factor, whereas Hull et al. (2008) found separate shifting and updating factors but no inhibition factor.

Inhibition in particular appears not to be a single construct but may tap different underlying inhibitory functions dependent on the tasks. Friedman and Miyake (2004) in a follow-up study of the inhibition factor found two separate inhibitory functions—inhibition of prepotent responses (and distractors), and resistance to proactive interference. Resistance to proactive interference has also been hypothesized to be a key problem in working memory tasks (e.g., May et al., 1999), and indeed Friedman and Miyake (2004) found that resistance to proactive interference predicted reading span. It is therefore likely that successful updating requires the ability to overcome proactive interference.

### Evidence from focal frontal lesion patients

Task-switching, updating and inhibition have also been identified in the clinical literature as frontal-based processes that are impaired differentially in patients with lesions to different regions of prefrontal cortex. Furthermore, although tasks requiring switching of task sets such as those used in the Miyake model are much simpler than tasks such as WCST, studies of individuals with focal frontal lesions suggest that even these simple tasks may require several executive processes depending on the precise demands of the task and the measures that one derives from them (e.g., Shallice et al., 2008; Stuss, 2011). Thus, correlations among latent variables in the statistical models may occur not because of some common overriding executive process but because even simple tasks are not process pure. Task-switching, for example, may require updating, monitoring, and inhibition as well as task-setting and switching.

Nevertheless, it is possible to separate some of these processes in focal lesion patients. In a review of the assessment methods for frontal lobe dysfunction, Stuss and Levine (2002) reported that dorsolateral prefrontal (DLPFC) lesions were preferentially associated with the set-shifting aspect of the WCST, whereas loss of set was evident in patients with ventrolateral prefrontal cortex (VLPFC) lesions, perhaps attributable to greater susceptibility to interference. In two more recent papers, Stuss (2011) and Stuss and Alexander (2007) identified two domain-general executive processes that are impaired in patients with dorsolateral frontal lesions—task-setting, which was defined as setting of stimulus-response contingencies in any task (left DLPFC), and monitoring of ongoing performance (right DLPFC). Although these processes do not map exactly onto the latent variable constructs from the factor analytic models, they nevertheless seem broadly consistent with or related to the processes of task-shifting, monitoring, and control of interference that were identified in those models. Stuss et al. also identified a process referred to as energization (which they did not describe as an executive process), which involved the initiation and sustaining of a response and was associated with superior medial prefrontal cortex. They found that patients with lesions in this region showed impairments in the incongruent condition of the Stroop task (and other reaction time (RT) tasks), which they attributed to a failure to sustain activation of the intended response.

### Evidence from neuroimaging

Neuroimaging studies have also found evidence consistent with multiple executive functions. For example, DLPFC has been associated with complex span tasks that require updating and monitoring of working memory (e.g., D'Esposito et al., 1999), whereas VLPFC has been associated with control of proactive interference and inhibition of prepotent responses (Jonides et al., 1998; D'Esposito et al., 2000). Task switching has been associated primarily with DLPFC, but VLPFC and parietal cortex have also been implicated (Braver et al., 2003), consistent with the idea that executive control functions involve a frontoparietal network. Sylvester et al. (2003) noted common areas of activation in several regions of prefrontal and parietal cortices for task switching and inhibition, as well as unique areas for each construct. Kim et al. (2011), in a recent study of cognitive flexibility, identified a domain-general switching mechanism that was localized to the inferior frontal junction (BA 44/6/9) and posterior parietal cortex (BA 7/40), and several domain-specific switching regions in medial and lateral prefrontal cortex (PFC) that varied as a function of the type of switching (cognitive set, stimulus or response). Finally, in a meta-analysis of neuroimaging studies of the WCST, task-switching and a go/no-go task, Buchsbaum et al. (2005) similarly reported a common frontal parietal network with specific sub-areas of PFC associated with task-switching (bilateral VLPFC, BA 47) and response inhibition (right DLPFC, BA 44/45/46). These findings, although not entirely consistent on the specific localization of different functions, nevertheless are fairly consistent in revealing distinctions between the constructs of shifting, updating and monitoring, and inhibition.

### Evidence from aging

Similar constructs have been examined in the aging literature but not all of them have been found to be age-sensitive. For example, age effects in task-switching have tended to be found in global switching but not in local switching (e.g., Verhaeghen and Cerella, 2002). Global switching costs reflect the increased time taken to complete a block of trials in which two tasks have to be performed in alternating or random order, compared to blocks of trials in which each of the tasks are performed separately. Local switching costs are measured as within-block time differences between switch trials and non-switch trials. Although both switches result in increased reaction times, the global cost seems to be most affected by age. This finding may be partly attributable to the extra costs of maintaining the two task sets in working memory.

Working memory tasks have generally shown substantial age effects particularly on the more complex span tasks, such as reading and operation span, which require updating and monitoring. These tasks also predict higher cognitive functions such as reasoning and problem solving (Kyllonen and Christal, 1990; Engle et al., 1999), episodic memory (Park et al., 1996), and fluid intelligence (see Kane et al., 2007) in both older and younger people. Age effects in measures of inhibition have not been found reliably in older adults, particularly on one of the most commonly used tests of inhibitory function, the Stroop (1935), although some studies have shown age-related deficits (e.g., Davidson et al., 2003). However, a recent study (Clark et al., 2012) reported that scores on a color-word interference test from the Delis-Kaplan Executive Function System (D-KEFS) were predictive of cognitive decline on the Dementia Rating Scale (Mattis et al., 1988) a year later, suggesting this test might be sensitive particularly to pathological aging (see also, Hutchison et al., 2010; Bayard et al., 2011). Older adults do show age-related deficits on tasks that require control of proactive interference (see, Hasher et al., 2007), and on stop-signal and go/no-go tasks as well as anti-saccade tasks (e.g., Nielson et al., 2002; Hasher et al., 2007), which have been associated with inhibition.

### Executive function tests

Although there is still considerable inconsistency in the literature, there is some consensus that there are at least three separable functions or processes, similar to those identified in Miyake et al. (2000)—task or set shifting, updating, and monitoring, and inhibition—which seem to depend partly on different regions of prefrontal cortex. In addition, inhibition may be further divided into two sub-components: inhibition of prepotent responses or distractors, and resistance to proactive interference. Complex tasks may require more than one of these processes. We therefore recommend that the executive function tasks used in cognitive aging be selected so as to tap into one of these identified functions, and that they be age-sensitive. We also suggest that one or more of the complex tasks that have been commonly reported in the literature be included for comparisons to previous studies and to potentially allow for a de-construction of the component processes underlying these tasks.

For each of the component processes, three tests that are related to the underlying process of interest should be selected. Ideally these tests should differ in other aspects so that the composite is most likely to reflect the targeted executive component. The following is a list of tests that have been associated with each specific function and appear to be sensitive to normal and/or pathological aging. Further details on the methodologies can be found in the papers referenced.

#### Shifting

- *Plus-minus task*: In this task, people see three separate lists of 30 two-digit numbers. For List1, they add 3 to each number; for List 2, they subtract 3 from each number; for List 3, they alternate addition and subtraction by 3. Shift cost is measured by the difference between time to complete List 3 and the average time for Lists 1 and 2 (from Miyake et al., 2000). This version of the task measures global shift costs. It may also be important that no external cues are provided (i.e., no + or − signs appear). Internally generated shifts appear to be more sensitive to aging than externally cued shifts.
- *Number-letter task*: In this task (Rogers and Monsell, 1995; Miyake et al., 2000; Gamboz et al., 2009), a number-letter pair (e.g., 8*F*) is presented in one of four quadrants. If the stimulus appears in the top two quadrants, participants make an odd/even judgment about the number; if it appears in the bottom two quadrants, they judge whether the letter is a consonant or vowel. In the first block of trials, stimuli are all on the top; in the second block they are all on the bottom, and in the third block stimuli shift in a clockwise fashion such that half of stimuli appear on the top, and half are on the bottom and a shift is required on half the trials. Both global and local shift costs can be calculated.
- *Global-local task*: In this task, people view a figure in which the lines of a global figure (e.g., a square) are constructed of smaller local figures (e.g., triangles). As in the previous tasks, participants are required to shift between the global and local figures, reporting how many lines make up the figure. Depending on how the task is constructed, one can measure global or local shift costs as described previously (Miyake et al., 2000).

Shifting tasks in humans may tap processes similar to those used in delayed alternation tasks in rodents, which show age deficits. In addition, the ability to shift attention from one perceptual dimension to another perceptual dimension of the same stimulus (i.e., an extra-dimensional shift) has been assessed with a variety of tasks in both rodents and non-human primates (see Bizon et al., 2012; also Table 1). In rodents, these tasks are dependent upon the rodent homologue of primate DLPFC and are analogous in design to the WCST.

**Table 1:** **Executive processes and associated tests for use in humans and animal models**.

**Executive process**	**Human tests**	**Animal tests**
Shifting	Plus-minus	Delayed alternation
	Letter-number	Extra-dimensional shift
	Global-local	
Updating/resistance to proactive interference	Consonant updating	Delayed matching to sample
	Keep track	
	Operation span	Delayed match-to-place
Inhibition of prepotent responses	Stroop	5-choice serial reaction time
	Simon	
	Go/NoGo	Stop signal

#### Updating/resistance to proactive interference

- *Letter memory/consonant updating*: In this task (Morris and Jones, 1990; Miyake et al., 2000; Vaughan and Giovanello, 2010), single letters are presented visually on a computer screen one at a time for 2 s each, and participants are required to repeat the last four letters out load, continually updating the working memory set, and then recall the last four consonants at the end of the list. List lengths vary randomly (5, 7, 9, and 11).
- *Keep track task*: Participants are first familiarized with category labels and instances of six categories (see Miyake et al., 2000). On each subsequent trial block, a subset of the category labels is shown at the bottom of the computer screen and remains visible throughout the trial block. Two to three instances from each of the six categories appear one at a time on the screen for 1500 ms, and participants monitor for the last word of each of the target categories. Miyake et al. (2000) used 15-item lists with 3 blocks of 4 and 5 target categories. In the modified version used by Hull et al. (2008) with older adults, four blocks of 10 trials were presented with only two target categories. We recommend using 3 blocks of 2, 3 and 4 categories with 15-item lists.
- *Operation span*: Operation span, although more complex than the other measures, is a classic working memory task developed by Turner and Engle (1989) in which people are asked to verify a solution for simple arithmetic problems [e.g., (2 × 4) −3 = 3] and retain a following word in memory (e.g., mouse). Set size varies from 2 to 5 problem-word pairs with three trials of each set size. After each set of problem-word pairs is presented, people recall the words in any order. Miyake et al. reported that his latent updating variable predicted performance on operation span (see also, Fisk and Sharp, 2004). Complex span tasks, including operation span, are sensitive not only to normal aging, but also in increasing fashion to mild cognitive impairment (MCI) and AD (Gagnon and Belleville, 2011).

Updating tasks in humans, which require resistance to proactive interference, appear similar to tasks described in Bizon et al. (2012; see also Table 1) that involve delayed matching to sample. For example, using a delayed-match-to-place version of the Morris water maze task, in which rats must learn a new platform location each day, older rats showed poorer delayed retention of the trial-unique stimuli, arguably because of greater interference from previous trials. Their performance on this task was unrelated to their performance on a spatial reference memory task that required them to remember the same platform location each day.

#### Inhibition of prepotent responses

- *Stroop task* (Stroop, 1935): There are several versions of the Stroop Color-Word Task. We recommend using a version that does not require task shifting, namely one in which the participants are always required to name the color of the ink. The key comparison is between the incongruent color-word and a neutral condition, which most often involves naming the color of asterisks or blocks of color. In some cases, a neutral word is used as an intermediate case. Both reaction time and errors should be recorded; the latter appear to be more sensitive to pathological aging (Hutchison et al., 2010). The measure commonly reported is the difference in RT between the incongruent and neutral conditions (Miyake et al., 2000), although a ratio of incongruent to neutral RTs has also been recommended to account for general slowing (e.g., Bayard et al., 2011).
- *Simon task* (Simon, 1990): In the Simon task, people are instructed to press the left button when a left-pointing arrow appears and the right button when a right-pointing arrow appears. The critical conditions occur when a right-pointing arrow occurs on the left of the screen or a left-pointing arrow occurs on the right of the screen, creating a mismatch between the location of the stimulus and the location of the response. Evidence suggests that the stimulus/response spatial match is the more primitive response, and has to be inhibited in order for a directional response to be made. The dependent measure is the RT difference between the congruent and the incongruent mapping. Castel et al. (2007) have found that this task is sensitive to normal aging and also distinguishes normal aging from mild AD.
- *Go/NoGo task*: Several versions of Go/NoGo tasks have appeared in the literature. We propose a task used by Nielson et al. (2002), which minimizes the working memory/updating component and emphasizes the inhibition component. The task involves continuous presentation every 500 ms of various letters of the alphabet with targets X and Y. Participants are required to respond with a button press to the occurrence of X and Y when they alternate in the serial sequence but not when they repeat consecutively (i.e., respond to X when it follows Y but not when it follows X). The alternating rule is added after two practice runs of “go” trials to establish the prepotent response. Letters are presented continuously at a 500 ms rate with a ratio of go to no go responses of between 3:1 and 6:1. The dependent measure is the percentage of successful inhibitory responses.

Several tasks (see Table 1) have been used in rodents to assess the ability to inhibit prepotent responses, including the five-choice serial reaction time task and the stop signal reaction time task (see Robbins et al., 2002; Eagle et al., 2008; Bizon et al., 2012). These tasks were developed to be analogous to tests of response inhibition in humans, and are sensitive to both damage to prefrontal cortex and advanced age (Winstanley et al., 2006; Harati et al., 2011).

#### Complex tasks

Even though it likely involves multiple executive processes, the WCST remains the gold standard of executive function tests. Although a measure of perseverative errors allows for a broad range of scores, not all studies have found perseverative errors to be age-sensitive. Additional measures—number of categories achieved, total errors, number of trials needed to achieve a category—should also be recorded and may reflect different component processes. The WCST has most often been associated with switching but as noted above, it may well involve other executive function processes as well.

## Memory

### Evaluating memory in older adults

Memory problems are one of the most common complaints among older adults. An extensive literature exists demonstrating that older adults are indeed impaired relative to young adults on some, but not all, types of memory. Most relevant to the present discussion of aging is the distinction between semantic and episodic memory (Tulving, 1972, 2002). There is ample evidence that episodic memory (memory for specific personal events that includes event-specific spatial and temporal context) declines over the adult lifespan while semantic memory (our general store of knowledge including words, concepts and facts about the world and ourselves) remains relatively stable across the adult lifespan (e.g., Nilsson et al., 2002). Those semantic tasks that decline are generally ones that require the rapid retrieval of information, such as category fluency (Nyberg et al., 2003), suggesting that older adults have problems related to efficient retrieval, rather than a deficit in semantic representations.

Several theoretical accounts of age-related memory changes have been proposed. Theoretical views have emphasized age-related declines in speed of mental processing (e.g., Park et al., 1996; Salthouse, 1996), decreases in general resources necessary for effortful processing (e.g., Craik, 1986; Craik and Rose, 2012), the inability to bind new associations among elements of an event (e.g., Chalfonte and Johnson, 1996; Naveh-Benjamin, 2000), decreases in inhibitory processing or executive functions (e.g., Hasher et al., 1999), limitations in working memory capacity (e.g., Cowan, 2010) and sensory-perceptual inefficiency (e.g., Baltes and Lindenberger, 1997). These views are not mutually exclusive, and they likely share a common outcome—inefficiency of processing, which leads to greater resources being expended during both encoding and retrieval, memory representations that are therefore less robust, and retrieval processes that are more prone to failure (Park and Reuter-Lorenz, 2009).

Memory deficits associated with aging are thought to be mediated primarily by two brain regions, medial temporal lobes and prefrontal cortex, which play different but interactive roles in memory, depending upon the specifics of the memory task. Although age-associated volume changes in the medial temporal lobes have been demonstrated (Small et al., 2002; Raz et al., 2005), aging disproportionately affects the prefrontal cortex compared with other brain regions (Raz and Rodrigue, 2006). Other age-related changes include the loss of integrity of white matter as measured by diffusion weighted imaging in both prefrontal and temporal regions (Ryan et al., 2011), reductions in dopamine production and dopamine receptors that are especially prominent in prefrontal cortex as well as medial temporal lobe regions (Giorgio et al., 2010), and amyloid deposition (Sperling et al., 2009).

Importantly, age-related memory impairments are not ubiquitous. There are considerable individual differences across older adults, and the source of these individual differences is of considerable interest to researchers. Likely they are determined by some combination of genetics, participant characteristics (e.g., verbal ability, education level, and domain expertise), developmental and environmental factors, health status (e.g., physical fitness, weight, and hypertension) and social and emotional variables (e.g., positive emotion and interpersonal goals).

The number and types of tests that have been used to assess memory in older adults are truly staggering, and include both traditional clinical neuropsychological tests and an extensive set of experimental measures. Here, we describe a small subset of these tasks that are well suited to assessing age-related memory functions, with sufficient sensitivity to capture not only age-related memory decline but individual differences in memory function among older adults. We will focus on four memory domains that have received considerable empirical attention: encoding and retrieval processes, associative, source, and prospective memory. Importantly, these processes and kinds of memory have all been shown to be modifiable in older adults by provision of environmental support or appropriate strategies, making them good targets for intervention.

### Memory domains

#### Encoding and retrieval

An influential view of aging and memory is the notion that the processing resources necessary for successful learning and remembering are available to a lesser degree in older adults compared to young adults (Craik, 1986, 2002). Craik (e.g., Craik and Byrd, 1982) originally conceptualized this impairment as a lack of available “mental energy,” which results in a failure to carry out self-initiated mental operations at both encoding and retrieval leading to poor memory. Thus, tasks that inherently require more self-initiated processing will be more likely to show age-related memory impairment. For example, lists of unrelated words are more difficult for some older adults to learn compared to stories or pictures. Similarly, tests with minimal retrieval cues such as free recall are more likely to show greater age-related decline than cued-recall and recognition (Craik and McDowd, 1987; Nyberg et al., 2003). This difference in performance has been attributed to older adults' dependence on familiarity-based rather than recollective retrieval processes (i.e., the dual-process model of recollection). Recall tasks, which are heavily dependent upon recollection, show greater age-related changes than familiarity-based tasks, such as recognition (Jennings and Jacoby, 1997; Bastin and Van der Linden, 2003), although there are considerable individual differences. For example, Davidson and Glisky (2002) showed that older adults with poor frontal function relied to a greater degree on familiarity and were therefore more prone to recognition errors.

#### Associative memory

One component of episodic memory decline in older adults is the degree to which they retain the ability to bind together the components of an episode (Mitchell et al., 2000; Naveh-Benjamin, 2000) or to associate items with their contextual features (Chalfonte and Johnson, 1996). Most commonly these tasks present previously unrelated pairs of items followed by independent tests of memory for the items and for the associations between the items. The consistent outcome of these experiments is a greater age deficit for associations relative to items. This pattern has been observed with word (Castel and Craik, 2003), word-spatial location (Mitchell et al., 2000), word-font (Naveh-Benjamin et al., 2003), name face (Naveh-Benjamin et al., 2004; Miller et al., 2008) and face-face pairs (Bastin and Van der Linden, 2003). Age-related impairment in paired associate learning has been related to reductions in hippocampal activity as well as dorsolateral prefrontal activity (Dennis and Cabeza, 2008). It appears that some portion of the associative deficit is likely mediated by an inability to produce appropriate strategies for learning and can be overcome to a great degree by providing older adults with specific strategies for remembering (Glisky et al., 2001; Naveh-Benjamin et al., 2007).

#### Source memory

**Figure 1:**
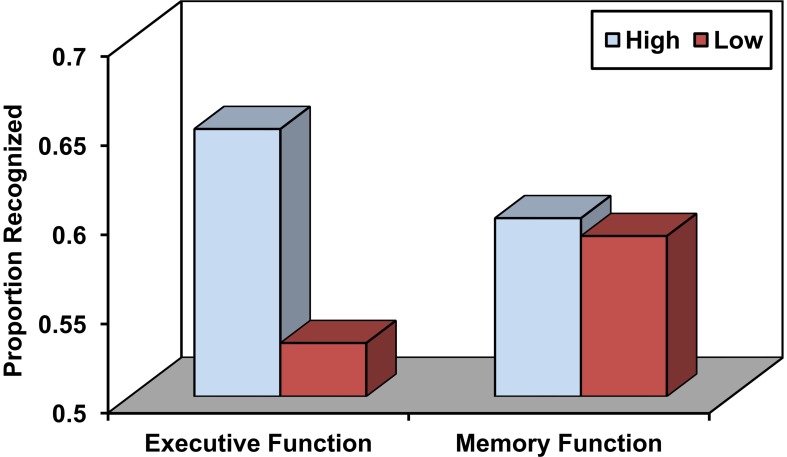
**Source memory in older adults as a function of high and low executive function and memory function.** Adapted from Glisky et al. (1995).

Source memory refers to the ability to remember the conditions surrounding the encoding of a particular episodic memory that may include information that specifies the source of the experience (e.g., Did you read it in the newspaper or in a magazine) as well as various aspects of the encoding context, including perceptual, spatio-temporal, affective, and other features (Johnson et al., 1993; Glisky and Kong, 2008). Source memory is particularly impaired in older adults relative to young adults (Chalfonte and Johnson, 1996; Mitchell et al., 2000; Glisky et al., 2001) and to a greater degree than item memory (Spencer and Raz, 1995). These deficits have often been associated with poor performance on frontally-mediated, executive function tests (see Figure 1; Glisky et al., 1995; Glisky and Kong, 2008) and less commonly with medial temporal lobe dysfunction (Schwerdt and Dopkins, 2001; Gold et al., 2006). Older adults also show decreases in fMRI activation relative to young adults in left ventrolateral prefrontal regions during short term source memory tasks, suggesting they may have specific problems comparing and evaluating information (Mitchell et al., 2006; for discussion see Glisky and Kong, 2008). One hypothesis is that reductions in frontal dopaminergic pathways may reduce the brain's ability to modulate incoming stimuli with respect to their specific contexts of occurrence, thereby resulting in less distinctive cortical representations of events that are difficult to differentiate from one another (Bäckman et al., 2000; Li et al., 2001).

#### Prospective memory

Remembering to perform an action at some future point is a memory skill that we use daily. In laboratory tasks of prospective memory (or “remembering to remember”), participants are engaged in some ongoing activity and need to monitor the environment for the presence of a cue. Upon recognizing the cue, they must recall its associated intention without any prompting and then interrupt their ongoing activity in order to successfully complete the intended action. In this way, prospective memory places a heavy burden on self-initiated processing. Two different types of prospective memory tasks have been studied. Event-based tasks refer to an intention that is associated with a particular cue (e.g., when you see the bank, deposit the check). Time-based tasks have no specific external cue, but rely instead on monitoring time (e.g., in 10 min from now, turn off the stove). A growing literature demonstrates that prospective memory is impaired in older adults, particularly those with poor executive function as measured by neuropsychological tests, for both event based tasks (McDaniel et al., 1999; McFarland and Glisky, 2011) and time based tasks (Martin et al., 2003; McFarland and Glisky, 2009).

When prospective memory fails, it can have tremendously negative consequences for older adults, particularly for those individuals who, for example, take multiple medications daily (Park et al., 1992; Insel et al., 2006). Prospective memory is an area that warrants additional research not only because of its theoretical implications, but because of the immediate and important application to quality of daily life.

### Memory measures

The majority of empirical studies on age-related memory impairment have utilized paradigms that are specific to a single laboratory, and often, to a single empirical study. The lack of standardized tasks makes direct comparisons across studies difficult, and combining data across sites impossible. The field would benefit greatly from the development of standardized versions of some of these paradigms. Some of the existing paradigms, particularly in the visual memory domain, have excellent analogs to standard tests utilized in animal models which are highlighted below.

#### Verbal encoding and retrieval

This is one area where well-developed neuropsychological measures of episodic memory are widely available. In the verbal domain, we favor the inclusion of memory tests that include both a list learning task and a story recall task in order to assess the differences between verbal materials that are unrelated and verbal materials that are embedded in a richly elaborated conceptual context (i.e., a story). Together, they assess memory for unrelated and related materials, the influence of external cues through comparisons of recall and recognition performance, and the differences between immediate recall and retention of information over a delay of 20–30 min.

- *Word lists*: List learning tasks involve the presentation of a list of words over multiple learning trials. Free recall is tested after each list presentation, and an overall learning score can be computed. Delayed free recall of the word list is typically obtained 20–35 min later, followed by yes–no recognition. The two most commonly used list learning tasks are the ***California Verbal Learning Test-2*** *(CVLT-2)* and the ***Rey Auditory Verbal Learning Test*** *(RAVLT)*. The CVLT-2 includes 16 words that belong to four different semantic categories, allowing measures of semantic organization during free and cued recall. The RAVLT, a list of 15 unrelated words, may provide a purer measure of encoding ability since good performance requires the participant to engage in elaborative and self-initiated processing.
- *Story memory* (Wechsler, 1987, 1997): Memory for semantically meaningful verbal material is generally assessed using short stories that are presented orally. Because of the structured nature of stories, there is minimal demand for self-initiated organization to link elements together. The most common clinical test with extensive age-related normative data is the Logical Memory subtest from the Wechsler Memory Scale (WMS). Generally, two different stories are read to the participant and an immediate free recall test is given for each. Delayed free recall for each story takes place approximately 30 min later followed by a multiple choice recognition test. Although the WMS is now in its fourth version and the stories and details of administration have changed, for studies with older adults, the WMS-R version (Wechsler, 1987) is ideal because of the simplicity of the design and administration, and norms available for all age groups. Alternative stories that are similar in characteristics and norms to the original stories are also available, greatly enhancing the ability to engage in longitudinal memory assessments (Schnabel, 2012).

#### Visual-spatial encoding and retrieval

While visual information is often tested with recognition (e.g., memory for faces), other visual memory tasks require drawing designs or geometric figures from memory after a brief exposure. The latter is somewhat problematic because it can be difficult to separate visual memory problems from constructional difficulties. Visual tasks that rely on recognition alone, some of which are described here, are direct analogs to those used in animal studies and are good candidates for cross-species studies of visual memory (see Table 2). The most common of these tests, including the delayed non-match to sample (Mishkin and Delacour, 1975) and spontaneous object recognition (Murray and Bussey, 1999) are reviewed in Burke et al. (2012).

- *Memory for faces* (WMS-III; Wechsler, 1997): A series of target faces are shown one at a time and people are asked to remember them. Both immediate and delayed memory is tested by showing targets intermixed with novel faces in a yes–no recognition task.
- *Visual reproductions* (WMS-III; Wechsler, 1997): Participants are shown novel designs and are asked to draw them immediately from memory after viewing them for 10 s. Delayed recall memory is tested after a 20–30 min delay.
- *Rey-Osterrieth complex figure* (Meyers and Meyers, 1995): This difficult visual spatial task requires that participants first copy a complicated geometric line drawing, and then draw the design from memory immediately after viewing it or after a brief 3 min delay. A delayed recall drawing is also obtained after 20–30 min. For the memory portions of the test, many different cognitive abilities are required for optimal performance including visual spatial ability, memory, attention, planning, and working memory (Duleya et al., 1993). Participants are not told beforehand that they will be asked to draw the figure from memory; the immediate recall condition is therefore incidental learning.
- *Doors and people*: Doors and People, normed for ages 18–80 (Baddeley et al., 1994), assesses visual recall and recognition using two types of stimuli that are difficult to encode verbally. The doors subtest assesses visual recognition by showing the participant a variety of different colored doors which they must remember and later recognize from a selection of similar doors. The shapes subtest assesses visual recall by asking the participant to copy four different patterns and then draw them from memory.

**Table 2:** **Human memory tests and promising analog tests currently utilized in animal models of memory**.

**Executive process**	**Human tests**	**Animal tests**
Visual recognition	Memory for faces, doors and people recognition	Spontaneous recognition Delayed nonmatch-to-sample
Spatial memory	Field and virtual reality spatial navigation tasks	Morris water maze Radial 8-arm maze
Associative memory	Visual paired associates, doors and people name-face pairs	Object-in-location recognition Contextual fear conditioning
Source memory	Visual task e.g., chairs in rooms	Delayed nonmatch-to-position
Prospective memory	Event-based and time-based	Not yet developed

Although standardized tests of spatial memory and spatial navigation for humans are not available, human paradigms derived from non-human visual spatial memory methods have been employed in the cognitive aging literature and are promising candidates for further cross-species research (reviewed in Foster et al., 2012). For example, Newman and Kaszniak (2000) developed a human analog of the Morris water maze (Morris et al., 1982) using a tent-like enclosure and identified age differences in performance of a spatial memory task. Similarly, Bohbot et al. (2002) reported a series of real-world spatial memory tests designed to be similar to tasks used in rats. One test was an analog of the Morris water maze; human subjects located a sensor (hidden under carpeting) while moving about a room. Another task resembled the 8-arm radial maze commonly used in rodent experiments (Bizon et al., 2012). Several computerized virtual-environment versions of the water maze have also been developed (Jacobs et al., 1997; Iaria et al., 2003) that are sensitive to age-related changes in performance (Moffat and Resnick, 2002; Jansen et al., 2010) and show strong correlations between real-space and virtual space versions of the same task (Nedelska et al., 2012).

#### Associative memory tasks

While associative memory tasks abound in the experimental cognitive aging literature, few standardized and normed associative memory tasks are available. The most common tasks used to assess associative memory in animals are contextual fear conditioning tasks (reviewed in Foster et al., 2012). Versions of objects-in-location recognition tasks (Bizon et al., 2012; Burke et al., 2012) may be most similar to human visual paired associate tasks, and are likely candidates for developing human-animal analogs.

- *Verbal paired associates learning* (WMS-III; Wechsler, 1997): Eight pairs of unrelated words (e.g., “trunk-arrow”) are presented to participants followed by cued recall. The list is repeated four times, with a final cued-recall test after a 30 min delay. Yes/no recognition is also assessed after the delay period for intact pairs interspersed with completely novel pairs. No recombined pairs are included as recognition lures.
- *Visual paired associates learning* (CANTAB^1^): This subtest assesses cued recall for pattern-location pairs. Boxes are displayed on a computer screen and are opened in a randomized order. One or more of them will contain a pattern. The patterns are then displayed in the middle of the screen, one at a time, and the participant must touch the box where the pattern was originally located. If the participant makes an error, the locations are repeated. Difficulty increases through the test by increasing the number of pattern-location pairs from one to eight.
- *Doors and People*: The battery, described earlier, includes a face-name paired associate learning test. This is a cued-recall task, where participants must remember and then subsequently recall the names of four different people, both immediately and after a delay.

#### Source memory tasks

Given the wealth of experimental information in this domain, this could be a particularly fruitful area for new standardized test development. Source tasks could also prove useful as a bridge between human memory and context-dependent memory studies in animals, particularly delayed nonmatch-to-position tasks (reviewed in Burke et al., 2012).

Generally, source memory experiments utilize a one-to-many mapping, that is, multiple words, objects, or sentences are experienced in one of two different contexts. The “many” refer to the to-be-remembered items while source refers to the association between the item and its context. Most often, item memory and source memory are tested with separate sets of materials, counterbalanced for order of testing. Importantly, both item and source are tested using a two-alternative choice recognition paradigm, in order to keep the memory task demands as similar as possible.

- *Verbal source test*: Glisky et al. (1995) presented participants with multiple sentences spoken aloud by two different readers, and asked them to judge how likely they were to hear the sentence on the radio. Source memory was tested in a two-alternative forced choice recognition test. Each of the sentences was spoken by both of the voices, and participants judged which voice had spoken the sentence during the study phase.
- *Visual source test*: In a later study, Glisky et al. (2001) created a visual analog to the sentence/voice source test. They presented participants with pictures of various office chairs that were photographed in two distinctive settings—a laboratory office cubicle and the department lounge. The same general procedure was followed. Two separate study lists were presented, one followed by a two-alternative choice recognition test for the chairs (distractors were novel chairs in the same setting), and the other list followed by a recognition test for the source (the same chair presented in both rooms).

#### Prospective memory tasks

As is the case with source memory, prospective memory tests have not been standardized. Here we describe two types of tests, both used in the laboratory setting, that could conceivably be developed for cross-species studies with humans and animals.

- *Event based prospective memory task*: A typical example of an event based task is found in McFarland and Glisky (2011). Young and older adults engaged in a multiple choice trivia game, presented on the computer screen, which tested their general world knowledge. At the same time, they were given a secondary task that required them to press a key whenever they saw the word “state” appear on the computer screen. Half the participants were given the typical read-only instructions (when you see the word “state,” press the key). Other participants were given implementation intention instructions (when I see the word “state,” I will press the key). The measures in this study were the number of times a participant accurately pressed the key when the target word appeared on the screen.
- *Time based prospective memory task*: McFarland and Glisky (2009) used a similar multiple choice trivia game as the primary task, presented on the computer screen with touch screen technology to record responses. For the secondary task, a clock icon was visible in the upper right hand corner of the screen, with two boxes just below it that were numbered “1” and “2.” Participants were instructed that every 5 min, they were to touch the 1 and 2 boxes in alternating order. To monitor time, they could touch the clock icon and the time elapsed since their last button press would appear. This study measured not only the number of prospective memory tasks completed, but also the proximity of the box presses to the elapsed time, as well as the pattern of clock monitoring. For example, one of the findings in this study was that young adults and older adults with high executive functioning (measured by neuropsychological tests) monitored the clock more often in the 1 min interval prior to their next scheduled button press, while low executive functioning older adults did not significantly increase their clock-monitoring in the final minute.

## Processing speed

The measurement of processing speed during the performance of mental tasks has been an important part of the longstanding effort to identify the origins of the observed differences in cognitive abilities over the lifespan (Deary et al., 2010). Tasks designed to measure mental speed typically involve the assessment of an individual's efficiency in completing a series of test items that require relatively low cognitive demands (e.g., perceptual matching). Tests of information processing speed have been widely used as measures sensitive to the cognitive effects of multiple neurological and psychiatric disorders, such as Alzheimer's disease, multiple sclerosis, traumatic brain injury, and depression (e.g., Alexander et al., 1994; Kail, 1998; Comijs et al., 2001; Bashore and Ridderinkhof, 2002; Denney et al., 2004). Information processing speed has been consistently shown to be an important correlate of age in cross-sectional studies and has also been identified as a major source of variance in the relation of other cognitive measures with age (Salthouse, 1993, 2000; Kail and Salthouse, 1994).

There is growing interest in characterizing the neural substrates of information processing speed and the neurobiological pathways that lead to age-related cognitive slowing with emerging support for the importance of the integrity of white matter tracts and the connectivity of regionally distributed brain networks (Rabbitt et al., 2007; Deary et al., 2009; Kennedy and Raz, 2009; Bartzokis et al., 2010; Penke et al., 2010; Burgmans et al., 2011; Eckert, 2011; Takeuchi et al., 2011; Lee et al., 2012).

Research is ongoing and new measures of information processing speed continue to emerge in the literature (e.g., Wiig et al., 2007) from both clinical/neuropsychological and cognitive/experimental approaches. The differences in their methods of acquisition, levels of stimulus complexity, and assessment of response timing suggest a potential complementary value for including tests represented by both classes of information processing measurement within a comprehensive battery for cognitive aging.

### Clinical/neuropsychological measures

The neuropsychological approach often requires decisions following the recognition, matching, or discrimination of perceptual stimuli, with accuracy providing a measure of performance within a specified time limit. The Coding and Symbol Search subtests of the Wechsler Adult Intelligence Scales – IV are two common examples of this class of processing speed measures that can be combined to form a composite score for a Processing Speed Index (Wechsler, 2008).

- *Coding (a.k.a. Digit-Symbol)*: Participants select and draw a visual symbol below a corresponding number derived from a digit-symbol code key presented at the top of the page. The score is based on the number of correct digit-symbol matches within the time limit.
- *Symbol search*: Participants perform multiple trials in which they place a line through two target symbols presented within a series of five possible choices. If no match is found, a line is drawn through a “No box.” The score reflects the number of correct responses across all test trials within a specified time.
- *Trail making test, Part A* (Trails A; Reitan, 1958): This test measures the time required to draw lines connecting a series of numbers within circles arranged non-contiguously on a page.
- *Letter and pattern comparison tests* (Salthouse and Meinz, 1995): These tests were developed specifically for studies of cognitive aging. Participants are required to make same versus different judgments on a series of letter strings or line patterns that contain three to nine line segments presented on pages with a specified time limit. The total number of correct responses minus the number of incorrect responses during the test time is calculated.
- *Finger-tapping* (Reitan and Wolfson, 1993): Participants are required to use their index finger to repeatedly press a key counter as quickly as they can in 10 s with their dominant and non-dominant hands. The total number of key presses with each hand during the time limit provides the finger tapping scores. An electronic version of the finger tapping test has been recently applied in a study of cognitive aging to provide a measure of simple motor processing speed that has been associated with neural function and myelin integrity (Bartzokis et al., 2010).

### Cognitive/experimental measures

The cognitive or experimental approach to measuring information processing speed has typically relied on computerized administration of tasks that assess component processes reflecting simple (SRT) and choice (CRT) reaction times. Although these tasks are varied in the types of stimuli and their complexity, they tend to present relatively simpler visual stimuli compared to the psychometric tests and measure average or median response times for individual task trials, rather than measures of performance integrated over a specified test time limit.

One example of a SRT task used in studies of cognitive aging requires subjects to press a button when a zero is presented on a screen using a variable 1–3 s inter-trial interval presented on a stand-alone, rectangular stimulus presentation/response box (Deary et al., 2001, 2010; Der and Deary, 2006; Penke et al., 2010). Response times are averaged for correct responses over the 20 test trials to obtain a measure of test performance. A corresponding four-choice reaction time task has been used in conjunction with this SRT task in which subjects are instructed to press one of four keys when the numbers 1, 2, 3, or 4 appear on the screen. The same 1–3 s variable inter-trial interval is used in this CRT task and test performance is measured by averaging response times for correct responses across 40 test trials. A freely available, computerized version of the SRT and CRT reaction time test has been recently developed, the Deary-Liewald reaction time task (Deary et al., 2011), that has shown high correlations with the established response-box SRT and CRT tasks.

Another example of an SRT test has been developed and applied in a recent study of aging in which subjects are instructed to touch a computer-screen, using a stylus, when they see a yellow square appear. The single visual stimulus is repeatedly presented using 1, 2, or 4-s inter-trial intervals in random order with 18 total test trials. A CRT version of this SRT task simultaneously presents subjects with two squares and requires them to touch the upper square if the two presented squares are the same color. They are required to touch the bottom square if the two presented squares are a different color. The dual visual stimuli are presented using the same 1, 2, or 4-s random inter-trial intervals with a total of 20 trials presented. Performance in both tasks is measured as the median hit response times over test trials (Lee et al., 2012).

An alternative experimental method to test processing efficiency has been proposed using a psychophysical approach (Deary et al., 2004). In this method, measures of visual inspection time are obtained as participants make discriminations between two parallel lines of different lengths presented on a computer screen by pressing one of two keys if the longer line is on the left or right side. Importantly, in this task, the subjects respond at their own pace with no response time recorded. The line pairs are each shown 10 times using 15 different durations extending from 6 to 200 ms. Since mean responses per duration length can range from chance level performance at the shortest presentations to nearly 100% accuracy at the longest presentations, the total number of correct responses provides an index of visual processing efficiency without measuring motor response speed (Deary et al., 2004). In a recent comparative study of several types of processing speed measures, the visual inspection time task was found to be least dependent on early life measures of overall intellectual ability, supporting its potential value as a complementary cognitive marker of processing speed during aging (Deary et al., 2010). This measure, however, may be more sensitive to administration differences, influences of ambient lighting, and degrees of intact or corrected peripheral visual processing.

Of the processing speed measures commonly used in human studies, the SRT and CRT tasks lend themselves particularly well to cross-species studies of aging and processing efficiency. For example, animal studies have examined reaction time using tasks analogous to SRT and CRT. Very little change in reaction time is observed between young adult (4–6 months) and aged (2 year old) rats on simple stimulus-response operant tasks (Menich and Baron, 1984; Burwell and Gallagher, 1993). However, an overall slowing of reaction time latencies is observed for animals older than 2 years. Moreover, there is considerable individual variability suggesting differences in biological aging.

For choice reaction time, a five-choice serial reaction time (5-CSRT) task has been employed (Harati et al., 2008). In this task the presentation of a light in one of five response openings signals where the animal needs to make a response (nosepoke) to receive a food reward. In addition, the stimulus duration can be varied (2–0.2 s). The latency to make a correct response to the light stimulus is a measure of decision-making speed, and latency between a correct response and food collection is used as a measure of motor function. An examination of age-related differences under standard conditions (0.5 s visual stimulus duration) revealed that decision-making speed was reduced in aged animals (25 months), compared to young adult and middle-aged rats. Decision-making speed continued to be reduced when a longer stimulus (2 s) was employed. Impaired processing efficiency, as measured by total number of correct responses, was reduced in aged animals when the stimulus was shortened (0.2 ms). The results suggest that a decline in simple and choice reaction time does not appear until late in life, and it is likely to be variable, depending on preceding life history, including cognitive and sensory-motor stimulation through environmental enrichment. For further details, see Bizon et al. (2012).

## Language

A widely accepted dictum is that vocabulary and other language-related skills remain relatively preserved during the course of normal aging. In this section, components of the language system (i.e., semantics, phonologic-orthographic) will be briefly described along with current data suggesting that while some aspects of language are *relatively* impervious to the effects of aging, others are not. Even those language components that remain relatively stable with age can become susceptible to other co-occurring cognitive changes outside the language system (i.e., working memory). We will briefly discuss views about “core vs peripheral” neural substrates of language, and findings over the past decade that neural networks supporting language differ in younger and older adults. Finally, an outline will be provided of commonly used language measures in clinical assessments along with a rationale for screening older adults who participate in studies of healthy cognitive aging.

### Components of language

The language system can be fractionated in multiple ways (Coltheart, 1987; Caplan, 1993; Caramazza and Mahon, 2006) but the fundamental components of language include a lexicon or vocabulary (words denoting objects, actions, and their modifiers), syntax (rules specifying relationships among words) and a phonologic-orthographic system (sounds and written symbols that constitute the actual spoken or written word). The term *lexico-semantic* typically refers to semantic meaning or knowledge that is conveyed by words, phrases, and syntax. *Naming* involves knowing what the object is (accessing semantic representation) and then accessing its phonologic representation in order to articulate that word. There are numerous cognitive information models that describe this process quite elegantly, and a variety of views on the breakdown of naming (Patterson and Shewell, 1987). A higher level aspect of language is pragmatics, which refers to how situational and contextual information can alter meaning of linguistic communication (i.e., inferred intent of speaker).

Another important distinction, drawing from the aphasia literature, is *comprehension* versus *speech production* (Goodglass and Kaplan, 1983). Speech output, or fluency, refers to ease and effort of articulation as well as quantity of output, and is associated with brain lesions anterior to the central sulcus (i.e., frontal) and can often involve Broca's area. Auditory comprehension deficits in aphasias are typically associated with posterior perisylvian lesions.

### Age-related constraints on language

At least three types of age-related phenomena influence language processing (Wingfield, 1996; Wingfield and Grossman, 2006). First, cognitive slowing results in older adults having more difficulty understanding rapidly presented speech (Wingfield et al., 1999). Second, reduced working memory capacity constrains the comprehension of syntactically complex sentences and results in less frequent use of complex syntax by older adults (Caplan et al., 2011). Kemper et al. (2001) analyzed a longitudinal corpus of speech samples obtained from older adults and found a marked decline in the grammatical complexity of the older adult's speech, which was directly related to working memory capacity. Third, the increased incidence of age-related hearing loss (particularly high frequency sounds) also affects comprehension of spoken language (Morrell et al., 1996). This can cause older adults to miss critical words during conversation, leading in some situations to reduced comprehension.

### Language skills affected by healthy cognitive aging

With aging, semantic aspects of language are generally well-maintained including vocabulary, language comprehension, and conversational discourse. Cross-sectional studies suggest that vocabulary increases with age until the 60s and 70s and remains stable thereafter (Verhaeghen, 2003; Salthouse, 2009). However, a somewhat different pattern emerges from at least two longitudinal studies that show age-related declines in vocabulary and other verbal skills starting around age 60 and continuing to decline more precipitously after age 74 (Berlin Aging Study, Baltes and Lindenberger, 1997; Seattle Longitudinal Study, Schaie, 2005). Even so, these language declines are less dramatic than those in other cognitive domains, such as episodic memory, and do not appear to undermine the functional adequacy of healthy older adults.

One of the most commonly experienced language-related problems across all ages is the inability to produce a well-known word on demand. These word retrieval difficulties become more frequent with age, and are evident on tasks of confrontation naming of object pictures (Kaplan et al., 1983; Burke et al., 1991; Au et al., 1995; Ivnik et al., 1996; Welch et al., 1996; Connor et al., 2004; Zec et al., 2005; Goral et al., 2007; Kave et al., 2010; see Feyerisen, 1997 for a meta-analysis). Similar age-related word retrieval problems are present on word generation tasks (verbal fluency) where individuals are required to rapidly produce words beginning with a target letter or from a semantic category (Troyer et al., 1997). One question is the extent to which these verbal fluency problems represent changes to language function, executive functions, or processing speed (McDowd et al., 2011). Another question relates to differential role of frontal and temporal lobe mechanisms in letter vs. category fluency tasks and how neural substrates for this distinction differ in young vs. old adults (Meinzer et al., 2009).

One prominent interpretation of age-related word finding difficulties is a decline in efficiency of lexical access, rather than degradation of verbal knowledge. Older adults produce more ambiguous references, have more filled pauses (um, er, etc.), and reformulate words more often than younger adults (Kemper et al., 2001; Burke and Shafto, 2004). These behaviors represent tactics for bypassing word retrieval difficulties. Access difficulties might stem from weakening of connections between a word's semantic and phonologic representations (Burke et al., 1991). It might also relate to age-associated changes in brain functions that affect frontally mediated search mechanisms (Weirenga et al., 2008; Meinzer et al., 2009), supported by functional MRI studies, diffusion tensor imaging, and connectivity analyses (Obler et al., 2010; Stamatakis et al., 2011).

### Core vs. support language networks

The classic aphasia literature identifies the left perisylvian cortical region as critical for language (Goodglass and Kaplan, 1983; Stemmer and Whitaker, 2008), including Wernicke's region within the temporal lobe, Broca's area within the frontal lobe, and connections between these two regions. More recent neuroimaging approaches have found that additional brain regions outside the perisylvian region also become activated during language processing—particularly anterior frontal regions as well as the homologous right hemisphere regions. Wingfield and Grossman (2006) draw a distinction between a core perisylvian language network that is necessary for language tasks, and additional regions beyond the core language network that become activated when healthy adults are engaged in language tasks. In line with this view, neuroimaging evidence indicates that older adults who successfully perform tasks such as naming and text processing recruit substantially more brain regions than poor performers or younger adults (Weirenga et al., 2008; Obler et al., 2010), including right hemisphere perisylvian and midfrontal regions, in conjunction with traditional left perisylvian regions (Obler et al., 2010). Those who are relatively poor namers do not recruit these support regions. This recruitment likely reflects compensation, which has been posited to account for findings of decreased laterality on various language-mediated cognitive tasks (Cabeza, 2002; Reuter-Lorenz and Park, 2010). One important implication of this line of research is that similar levels of performance accuracy on language tasks by younger and older adults may be mediated by different (though overlapping) neural circuitries.

### Translational challenges

Although there is little disagreement that animals have unique communication systems, considerable controversy exists regarding whether propositional language is exhibited by non-human primates or other animal species. While this controversy is beyond the scope of the current paper, suffice to say that few animal models of language currently exist. One line of animal research has focused on the basic neuroanatomic substrates of language, and whether non-human primates exhibit language-related brain asymmetries similar to those observed in humans. Geschwind and Levitsky (1968) were the first to document that the planum temporale, a region well known for its role in language comprehension, was larger in the left hemisphere than the right in humans. This asymmetry has been systematically replicated in postmortem and structural imaging studies (Steinmetz et al., 1989; Good et al., 2001; Eckert et al., 2006). Chimpanzees show a similar leftward asymmetry in the size of planum temporale, whereas lower monkey species (rhesus, vervet, and bonnet macaques) do not (Lyn et al., 2011).

### Assessment of language in older adults: rationale and recommendation

Relative to other cognitive domains (executive function, processing speed, and episodic memory), language is less sensitive to the influence of underlying age-associated neural changes (Raz et al., 2010). Even so, commonly occurring neurodegenerative conditions, such as Alzheimer's disease, typically disrupt aspects of language, particularly word retrieval and naming. These changes occur secondary to alteration of temporo-parietal networks involved in semantics. In fact, impairments on relatively simple tasks of confrontation naming and word generation, especially semantic fluency, are commonly observed in individuals with early Alzheimer's disease. Subtle deficits can even be seen in individuals with the amnestic variant of MCI, who are at greater risk for transitioning to dementia. Thus, language screening is particularly important if one's goal is to study memory or executive function in healthy older adults in the absence of co-occurring neurodegenerative disease.

Table 3 depicts an overview of commonly used measures in the clinical assessment of language in older adults, ranging from tasks of vocabulary knowledge, word retrieval (confrontation naming) and verbal fluency to those examining discourse and pragmatics. This table does not include batteries for assessing individuals with acquired aphasia that is sometimes associated with focal strokes but also observed in certain neurodegenerative conditions (i.e., semantic dementia, progressive non-fluent aphasia). Certain measures, particularly confrontation naming and speeded verbal fluency tasks, can be helpful for identifying subsets of older adults who may already be experiencing neurodegenerative changes. For this reason, many centers and large-scale studies routinely include a confrontation naming task, such as the Boston Naming Test (Kaplan et al., 1983), as one of several screening tools for the purpose of excluding (or including) individuals with early signs of dementia. The Boston Naming Test is relatively brief, consisting of 60 line drawings of items that an individual “names.” Short forms are available (30 and 15 item versions) and can be used rather easily for screening purposes.

**Table 3:** **Commonly used language tasks in clinical assessment of non-aphasic older adults**.

**Domain**	**Tasks**	**Older adult norms**
Semantics		
Knowledge	Expressive vocabulary (WAIS, WASI)	Yes
	Receptive vocabulary (PPVT)	Yes
Word retrieval	Visual confrontation naming (Boston naming test) Auditory confrontation naming	Heaton and MOANS
	Action naming	
Directed fluency	Letter fluency (COWA)	Heaton and MOANS
	Category fluency (animals, fruits-vegetables, etc.)	Heaton and MOANS
	Fluency tasks from DKEFS	Yes
Syntax comprehension	Token test (multilingual aphasia exam)	MOANS
Discourse	Oral description of complex pictures (i.e., cookie theft picture from BDAE, kite picture from the WAB) Open ended script questions	

Task Abbreviations: WAIS, Wechsler Adult Intelligence Scale; WASI, Wechsler Abbreviated Scale of Intelligence; PPVT, Peabody Picture Vocabulary Test; COWA, Controlled Oral Word Association Test; DKEFS, Delis-Kaplan Executive Function System; BDAE, Boston Diagnostic Aphasia Exam; WAB, Western Aphasia Battery.

Norms Abbreviation: Heaton refers to norms published in Heaton et al. (2004); MOANS refers to Mayo Older Adult Norms (MOANS), in Ivnik et al. (1996). See Lezak et al. (2012) for detailed description of individual test measures.

## Visuospatial functions

Visuospatial performance generally declines with age in both humans and other species (Studzinski et al., 2006). A good working definition of visuospatial dysfunction is attributed to Boller et al. (1984):

“Difficulty in appreciating the position of stimulus-objects in space, difficulty in integrating those objects into a coherent spatial framework, and difficulty in performing mental operations involving spatial concepts.”

There are many approaches intended to test human visuospatial performance. Most have grown out of clinical neuropsychology research with humans, rather than from the perspectives of experimental approaches with either humans or in animal models (but see a discussion of recent human and animal research on object discrimination in Burke et al., 2012). The field is further complicated by ongoing reconsideration of the basic organization of the neural and cognitive psychology of systems involved in responding to visual stimuli (Kravitz et al., 2011).

A major challenge to systematic study of this construct lies in the wide variety of cognitive functions subsumed under “visuospatial function.” Visuospatial tests might preferentially weight spatial perception and localization, spatial memory, and organization of cognitive and motor responses to spatial information. Aspects of visual perception affect performance on many visuospatial tasks and vary by instrument and presentation medium.

Aspects of the testing procedures related to other cognitive functions, rather than either visual or spatial processing losses, are significant contributors to age-related decline in visuospatial performance (Libon et al., 1994). For instance, when Kemps and Newsom (2006) used the “Doors and People test” to assess age-related changes in visual and verbal memory, they found that working memory and executive function made the greatest contributions to age related declines, while processing speed and perceptual issues were lesser contributors. Many of the tests considered to assess visuospatial abilities more closely map on to memory processes. In general these tasks demonstrate low reliability and limited construct validity (Moye, 1997). Even tests that clearly map to similar visuoperceptual processes such as the Gollin Incomplete Figures test and the Mooney test of Incomplete Faces Perception, show important variability in test samples, such as gender based differences in performance (Foreman, 1991). Overall, older adults appear to be vulnerable to the effects of reduced stimulus redundancy, decreased signal-to-noise, and limited processing speed. Offsetting those vulnerabilities by varying the testing procedure (e.g., altering stimulus exposure time or signal-to-noise ratio) attenuates, but does not eliminate age differences on relatively “pure” tests of visuospatial integration such as incomplete figure identification (Kennedy et al., 2009). Nonetheless, age can influence performance on several of these tests.

- The *Rey-Osterreith complex figure* test typically consists of three test conditions: Copy, Immediate Recall and Delayed Recall. Whereas the memory aspects of the test have been discussed previously, the copy portion tests graphomotor spatial functions; the subject is asked to draw a complex geometric line drawing. Age norms have been published, based on a sample of 211 adults ranging from 30 to 85, for the standard administration as well as supplemental matching trials (Fastenau et al., 1999). Matching may be more easily translated to studies of non-human species.
- The *Gollin incomplete figures* test requires subjects to identify a series of fragmented line drawings over successive stimulus presentations that increase the “completeness” of the drawings with age-decade cohorts (50–99, 60–69, and 70–79) showing declining performance (Read, 1988).
- The *Poppelreuter-Ghent* figures assess visual perceptual functions by asking subjects to identify overlapping line-drawing images. Performance of healthy controls is influenced by age and education, but not sex and normative values adjusted for age and education are available (Della Sala et al., 1995).
- The *Visual object and space perception battery* is a proprietary battery consisting of eight untimed tests, each designed to assess a particular aspect of object or space perception, while minimizing the involvement of other cognitive skills. Four subtests measure visual object perception (Incomplete Letters, Silhouettes, Object Decision, and Progressive Silhouettes). The other four measure visual space perception (Dot Counting, Position Discrimination, Number Location, and Cube Analysis). Herrera-Guzmán et al. (2004) reported significant age-cohort effects in 5 of the 8 subtests in a Spanish language sample. For the object perception tasks, age effects were significant for 3 of 4 subtests. For the space perception tasks, significant age effects were identified for 2 subtests. This confirms an earlier pattern seen in an American sample (Bonello et al., 1997). However, longitudinal data among elderly subjects has not been reported.

A major problem in aligning visuospatial assessment in humans and non-human species is that human tests typically require verbal output or higher-order cognitive tasks like manual drawing, whereas non-human species respond with whole body positions (e.g., moving to a target location) or simple manual responses (e.g., lever press). Also, the non-human testing is heavily biased toward learning and memory of visuospatial material, rather than action based on contemporaneously available visual information (see Burke et al., 2012).

## Summary

In this review, we presented a selected set of methods and approaches that have been applied in evaluating the effects of healthy cognitive aging on higher-order cognitive processes. We focused on five major cognitive domains, including executive function, memory, processing speed, language, and visuospatial function, each showing support for well-established age effects, while also presenting aspects of those domains that can remain relatively preserved with age. Examples from cognitive/experimental and clinical/neuropsychological approaches were presented, with a consideration of the measures that have clear linkages to tasks currently in use in animal studies of aging, as well as those that have the potential, with further development, to be translated for non-human animal aging research.

Using multiple measures both within and across cognitive domains can provide both task-specific and composite scores to identify the characteristic profiles associated with healthy cognitive aging. Such profiles may advance cross-sectional, longitudinal, and intervention studies with the potential for applications across species in the study of cognitive aging. As part of a targeted, rational approach to identifying effective interventions and prevention therapies, cognitive measures that can translate across species offers a unique opportunity to efficiently identify the most promising interventions for the effects of cognitive aging in rodents or in other non-human animal models prior to initiating much larger and more expensive human clinical trials. Further research is needed to expand the availability of a wide variety of complementary methodological tools to help advance cognitive aging research. Such research efforts may lead to a greater understanding of the underlying neural mechanisms with the potential for developing effective interventions and prevention therapies to slow, delay, or diminish the effects of age-related cognitive decline.

### Conflict of interest statement

The authors declare that the research was conducted in the absence of any commercial or financial relationships that could be construed as a potential conflict of interest.

## Footnotes

^1^The Cambridge Neuropsychological Test Automated Battery (CANTAB; Cambridge Cognition, http://www.cambridgecognition.com) was developed to provide human versions of tests that were widely used to test animal models of memory, making them particularly well-suited to cross-species studies of memory and aging (Robbins et al., 1994).

## References

[B1] Alexander G. E. Prohovnik I. Stern Y. Mayeux R. (1994). WAIS-R subtest profile and cortical perfusion in Alzheimer's disease. Brain Cogn. 24, 24–43 10.1006/brcg.1994.1002 8123262

[B2] Au R. Joung P. Nicholas M. Obler K. L. Kass R. Albert M. (1995). Naming ability across the adult lifespan. Aging Cogn. 2, 300–311

[B3] Bäckman L. Ginovart N. Dixon R. A. Robins T. B. Wahlin A. Halldin C. Farde L. (2000). Age-related cognitive deficits mediated by changes in the striatal dopamine system. Am. J. Psychiatry 157, 635–637 10.1176/appi.ajp.157.4.635 10739428

[B4] Baddeley A. D. Emslie H. Nimmo-Smith I. (1994). Doors and People: A Test of Visual and Verbal Recall and Recognition. Flempton, Bury St. Edmunds: Thames Valley Test Company

[B5] Baltes P. B. Lindenberger U. (1997). Emergence of a powerful connection between sensory and cognitive functions across the adult life span: a new window to the study of cognitive aging? Psychol. Aging 12, 12–21 9100264 10.1037//0882-7974.12.1.12

[B6] Bartzokis G. Lu P. H. Tingus K. Mendez M. F. Richard A. Peters D. G. Oluwadara B. Barrall K. A. Finn J. P. Villablanca P. Thompson P. M. Mintz J. (2010). Lifespan trajectory of myelin integrity and maximum motor speed. Neurobiol. Aging 31, 1554–1562 10.1016/j.neurobiolaging.2008.08.015 18926601 PMC2888859

[B7] Bashore T. R. Ridderinkhof K. R. (2002). Older age, traumatic brain injury, and cognitive slowing: some convergent and divergent findings. Psychol. Bull. 128, 151–198 11843546 10.1037/0033-2909.128.1.151

[B8] Bastin C. Van der Linden M. (2003). The contribution of recollection and familiarity to recognition memory: a study of the effects of test format and aging. Neuropsychology 17, 14–24 12597069

[B9] Bayard S. Erkes J. Moroni C. (2011). Victoria Stroop test: normative data in a sample group of older people and the study of their clinical applications in the assessment of inhibition in Alzheimer's disease. Arch. Clin. Neuropsychol. 26, 653–661 10.1093/arclin/acr053 21873625

[B10] Bell-McGinty S. M. Podell K. P. Franzen M. Baird A. D. Williams M. J. (2002). Standard measures of executive function in predicting instrumental activities of daily living in older adults. Int. J. Geriatr. Psychiatry 17, 828–834 10.1002/gps.646 12221656

[B11] Bizon J. L. Foster T. C. Alexander G. E. Glisky E. L. (2012). Characterizing cognitive aging of working memory and executive function in animal models. Front. Aging. Neurosci. 4:19 10.3389/fnagi.2012.00019 PMC3439637 22988438

[B12] Bohbot V. D. Jech R. Růžička E. Nadel L. Kalina M. Stepánková K. Bures J. (2002). Rat spatial memory tasks adapted for humans: characterization in subjects with intact brain and subjects with medial temporal lobe lesions. Physiol. Res. 51, S49–S64 12479786

[B13] Boller F. Passafiume D. Keefe N. C. Rogers K. Morrow L. Kim Y. (1984). Visuospatial impairment in Parkinson's disease. Role of perceptual and motor factors. Arch. Neurol. 41, 485–490 6721713 10.1001/archneur.1984.04050170031011

[B14] Bonello P. J. Rapport L. J. Millis S. R. (1997). Psychometric properties of the visual object and space perception battery in normal older adults. Clin. Neuropsychol. 11, 436–442 10.1076/jcen.20.2.211.1169 9777475

[B15] Braver T. S. Reynolds J. R. Donaldson D. I. (2003). Neural mechanisms of transient and sustained cognitive control during task switching. Neuron 39, 713–726 10.1016/S0896-6273(03)00466-5 12925284

[B16] Braver T. S. West R. L. (2008). Working memory, executive processes, and aging, in Handbook of Aging and Cognition, 3rd Edn., eds Craik F. I. Salthouse T. L. (New York, NY: Lawrence Erlbaum Associates), 311–372

[B17] Buchsbaum B. R. Greer S. Chang W.-L. Berman K. F. (2005). Meta-analysis of neuroimaging studies of the Wisconsin card-sorting task and component processes. Hum. Brain Mapp. 25, 35–45 10.1002/hbm.20128 15846821 PMC6871753

[B18] Burgmans S. Gronenschild E. H. B. M. Fandakova Y. Shing Y. L. van Boxtel M. P. J. Vuurman E. F. P. M. Uylings H. B. M. Jolles J. Raz N. (2011). Age differences in speed of processing are partially mediated by differences in axonal integrity. Neuroimage 55, 1287–1297 10.1016/j.neuroimage.2011.01.002 21232618 PMC3057324

[B19] Burke D. M. MacKay D. G. Worthley J. S. Wade E. (1991). On the tip of the tongue: what causes word finding failures in you and older adults? J. Mem. Lang. 30, 542–579

[B20] Burke D. M. Shafto M. A. (2004). Aging and language production. Curr. Dir. Psychol. Sci. 13, 21–24 10.1111/j.0963-7214.2004.01301006.x 18414600 PMC2293308

[B21] Burke S. N. Ryan L. Barnes C. A. (2012). Characterizing cognitive aging of recognition memory and related processes in animal models and in humans. Front. Aging. Neurosci. 4:15 10.3389/fnagi.2012.00015 PMC3439640 22988437

[B22] Burwell R. D. Gallagher M. (1993). A longitudinal study of reaction time performance in Long-Evans rats. Neurobiol. Aging 14, 57–64 8450934 10.1016/0197-4580(93)90023-5

[B23] Cabeza R. (2002). Hemispheric asymmetry reduction in older adults: the HAROLD model. Psychol. Aging 17, 85–100 11931290 10.1037//0882-7974.17.1.85

[B24] Caplan D. (1993). Language: Structure, Function, and Disorders. Cambridge, MA: MIT Press

[B25] Caplan D. Dede G. Waters G. Michaud J. Tripodis Y. (2011). Effects of age, speed of processing and working memory on comprehension of sentences with relative clauses. Psychol. Aging 26, 439–450 10.1037/a0021837 21480714

[B26] Caramazza A. Mahon B. Z. (2006). The organization of conceptual knowledge in the brain: the future's past and some future directions. Cogn. Neuropsychol. 23, 13–38 10.1080/02643290542000021 21049320

[B28] Castel A. D. Balota D. A. Hutchison K. A. Logan J. M. Yap M. J. (2007). Spatial attention and response control in healthy younger and older adults and individuals with Alzheimer's disease: evidence for disproportionate selection impairments in the Simon task. Neuropsychology 21, 170–182 10.1037/0894-4105.21.2.170 17402817

[B29] Castel A. D. Craik F. I. (2003). The effects of aging and divided attention on memory for item and associative information. Psychol. Aging 18, 873–885 10.1037/0882-7974.18.4.873 14692872

[B30] Chalfonte B. L. Johnson M. K. (1996). Feature memory and binding in young and older adults. Mem. Cogn. 24, 403–416 8757490 10.3758/bf03200930

[B31] Clark L. R. Schiehser D. M. Weissberger G. H. Salmon D. P. Delis D. C. Bondi M. W. (2012). Specific measures of executive function predict cognitive decline in older adults. J. Int. Neuropsychol. Soc. 18, 118–127 10.1017/S1355617711001524 22115028 PMC3314335

[B32] Coltheart M. (1987). Functional architecture of the language processing system, in The Cognitive Neuropsychology of Language, eds Coltheart M. Sartori G. Job R. (Hillsdale, NJ: Lawrence Erlbaum), 67–79

[B33] Comijs H. C. Jonker C. Beekman A. T. Deeg D. J. (2001). The association between depressive symptoms and cognitive decline in community-dwelling elderly persons. Int. J. Geriatr. Psychiatry 16, 361–367. 11333422 10.1002/gps.343

[B34] Connor L. T. Obler L. K. Albert M. L. Spiro A. III (2004). Change in object naming ability during adulthood. J. Gerontol. Psychol. Sci. 59, 203–209 15358792 10.1093/geronb/59.5.p203

[B35] Cowan N. (2010). The magical mystery four: how is working memory capacity limited, and why? Curr. Dir. Psychol. Sci. 19, 51–57 20445769 10.1177/0963721409359277 PMC2864034

[B36] Craik F. I. M. (1986). A functional account of age differences in memory, in Human Memory and Cognitive Capabilities: Mechanisms and Performances, eds Klix F. Hagendorf H. (North-Holland: Elsevier Science Publishers, B.V.), 409–422

[B37] Craik F. I. M. (2002). Human memory and aging, in Psychology at the Turn of the Millennium, eds Bäckman L. von Hofsten C. (Hove, UK: Psychology Press), 261–280

[B38] Craik F. I. M. Byrd M. (1982). Aging and cognitive deficits: the role of attentional resources, in Aging and Cognitive Processes, eds Craik F. I. M Trehub S. E. (New York, NY: Plenum Press), 191–211

[B39] Craik F. I. M. McDowd J. M. (1987). Age differences in recall and recognition. J. Exp. Psychol. Learn. Mem. Cogn. 13, 474–479

[B40] Craik F. I. Rose N. S. (2012). Memory encoding and aging: a neurocognitive perspective. Neurosci. Biobehav. Rev. 36, 1729–1739 10.1016/j.neubiorev.2011.11.007 22155274

[B41] Daffner K. R. (2010). Promoting successful cognitive aging: a comprehensive review. J. Alzheimers Dis. 19, 1101–1122 10.3233/JAD-2010-1306 20308777 PMC3047597

[B42] Davidson D. J. Zacks R. T. Williams C. C. (2003). Stroop interference, practice, and aging. Aging. Neuropsychol. Cogn. 10, 85–98 10.1076/anec.10.2.85.14463 PMC1761647 17203134

[B43] Davidson P. S. Glisky E. L. (2002). Neuropsychological correlates of recollection and familiarity in normal aging. Cogn. Affect. Behav. Neurosci. 2, 147–186 12455684 10.3758/cabn.2.2.174

[B44] Deary I. J. Corley J. Gow A. J. Harris S. E. Houlihan L. M. Marioni R. E. Penke L. Rafnsson S. B. Starr J. M. (2009). Age-associated cognitive decline. Br. Med. Bull. 92, 135–152 10.1093/bmb/ldp033 19776035

[B45] Deary I. J. Der G. Ford G. (2001). Reaction times and intelligence differences: a population-based cohort study. Intelligence 29, 389–399

[B46] Deary I. J. Johnson W. Starr J. M. (2010). Are processing speed tasks biomarkers of cognitive aging? Psychol. Aging 25, 219–228 10.1037/a0017750 20230141

[B47] Deary I. J. Liewald D. Nissan J. (2011). A free, easy-to-use, computer-based simple and four-choice reaction time programme: the Deary-Liewald reaction time task. Behav. Res. 43, 258–268 10.3758/s13428-010-0024-1 21287123

[B48] Deary I. J. Simonotto E. Meyer M. Marshall A. Marshall I. Goddard N. Wardlaw J. M. (2004). The functional anatomy of inspection time: an event-related fMRI study. Neuroimage 22, 1466–1479 10.1016/j.neuroimage.2004.03.047 15275904

[B49] Della Sala S. Laiacona M. Spinnler H. Trivelli C. (1995). Poppelreuter-Ghent's overlapping figures test: its sensitivity to age, and its clinical use. Arch. Clin. Neuropsychol. 10, 511–534 10.1016/0887-6177(94)00049-V 14588906

[B50] Denney D. R. Lynch S. G. Parmenter B. A. Horne N. (2004). Cognitive impairment in relapsing and primary progressive multiple sclerosis: mostly a matter of speed. J. Int. Neuropsychol. Soc. 10, 948–956 15803558 10.1017/s1355617704107030

[B51] Dennis N. A. Cabeza R. (2008). Neuroimaging of healthy cognitive aging, in The Handbook of Aging and Cognition, 3rd Edn., eds Craik F. I. M. Salthouse T. A. (New York, NY: Psychology Press), 1–54

[B52] Der G. Deary I. J. (2006). Age and sex differences in reaction time in adulthood: results from the United Kingdom health and lifestyle study. Psychol. Aging 21, 62–73 10.1037/0882-7974.21.1.62 16594792

[B53] D'Esposito M. Postle B. R. Ballard D. Lease J. (1999). Maintenance versus manipulation of information held in working memory: an event-related fMRI study. Brain Cogn. 41, 66–86 10.1006/brcg.1999.1096 10536086

[B54] D'Esposito M. Postle B. R. Rypma B. (2000). Prefrontal cortical contributions to working memory: evidence from event-related fMRI studies. Exp. Brain Res. 133, 3–11 10.1007/s002210000395 10933205

[B55] Duleya J. F. Wilkins J. W. Hamby S. L. Hopkins D. G. Burwell R. D. Barry N. S. (1993). Explicit scoring criteria for the Rey-Osterrieth and Taylor complex figures. Clin. Neuropsychol. 7, 29–38

[B57] Eagle D. M. Bari A. Robbins T. W. (2008). The neuropsychopharmacology of action inhibition: cross-species translation of the stop-signal and go/no-go tasks. J. Psychopharmacol. 199, 439–456 10.1007/s00213-008-1127-6 18542931

[B58] Eckert M. Leonard C. M. Possing E. T. Binder J. R. (2006). Uncoupled leftward asymmetries for planum morphology and funcational language processing. Brain Lang. 98, 102–111 10.1016/j.bandl.2006.04.002 16697453 PMC1661833

[B59] Eckert M. A. (2011). Slowing down: age-related neurobiological predictors of processing speed. Front. Neurosci. 5, 1–13 10.3389/fnins.2011.00025 21441995 PMC3061488

[B60] Engle R. W. Tuholski S. W. Laughlin J. E. Conway A. R. A. (1999). Working memory, short-term memory and general fluid intelligence: a latent variable approach. J. Exp. Psychol. Gen. 128, 309–331 10513398 10.1037//0096-3445.128.3.309

[B61] Fastenau P. S. Denburg N. L. Hufford B. J. (1999). Adult norms for the Rey-Osterrieth complex figure test and for supplemental recognition and matching trials from the extended complex figure test. Clin. Neuropsychol. 13, 30–47 10.1076/clin.13.1.30.1976 10937646

[B62] Feyerisen P. (1997). A meta-analytic procedure shows an age-related decline in picture naming. Comments on Goulet, Ska, and Khan (1994). J. Speech Lang. Hear. Res. 30, 1328–1333 10.1044/jslhr.4006.1328 9430752

[B63] Fisk J. E. Sharp C. A. (2004). Age-related impairment in executive functioning: updating, inhibition, shifting, and access. J. Clin. Exp. Neuropsychol. 26, 874–890 10.1080/13803390490510680 15742539

[B64] Foreman N. (1991). Correlates of performance on the Gollin and Mooney tests of visual closure. J. Gen. Psychol. 118, 13–20 10.1080/00221309.1991.9711129 2037842

[B65] Foster T. C. DeFazio R. A. Bizon J. L. (2012). Characterizing cognitive aging of spatial and contextual memory in animal models. Front. Aging Neurosci. 4:12 10.3389/fnagi.2012.00012 PMC3439636 22988436

[B66] Friedman N. P. Miyake A. (2004). The relations among inhibition and interference control functions: a latent-variable analysis. J. Exp. Psychol. Gen. 133, 101–135 10.1037/0096-3445.133.1.101 14979754

[B67] Gagnon L. G. Belleville S. (2011). Working memory in mild cognitive impairment and Alzheimer's disease: contribution of forgetting and predictive value of complex span tasks. J. Neuropsychol. 25, 226–236 10.1037/a0020919 21090897

[B68] Gamboz N. Borella E. Brandimonte M. A. (2009). The role of switching, inhibition and working memory in older adults' performance in the Wisconsin Card Sorting Test. Aging Neuropsychol. Cogn. 16, 260–284 10.1080/13825580802573045 19105052

[B69] Geschwind N. Levitsky W. (1968). Human brain – Left-right asymmetries in temporal speech region. Science 161, 186–187 10.1126/science.161.3837.186 5657070

[B70] Giorgio A. Santelli L. Tomassini V. Bosnell R. Smith S. De Stefano N. Johansen-Berg H. (2010). Age-related changes in grey and white matter structure throughout adulthood. Neuroimage 51, 943–951 10.1016/j.neuroimage.2010.03.004 20211265 PMC2896477

[B71] Glisky E. L. Kong L. L. (2008). Do young and older adults rely on different processes in source memory tasks? A neuropsychological study. J. Exp. Psychol. Learn. Mem. Cogn. 34, 809–822 10.1037/0278-7393.34.4.809 18605870 PMC2504728

[B72] Glisky E. L. Polster M. R. Routhieaux B. C. (1995). Double dissociation between item and source memory. Neuropsychology 9, 229–235

[B73] Glisky E. L. Rubin S. R. Davidson P. S. R. (2001). Source memory in older adults: an encoding or retrieval problem? J. Exp. Psychol. Learn. Mem. Cogn. 27, 1131–1146 11550742 10.1037//0278-7393.27.5.1131

[B74] Gold J. J. Smith C. N. Bayley P. J. Shrager Y. Brewer J. B. Stark C. E. L. Hopkins R. O. Squire L. R. (2006). Item memory, source memory, and the medial temporal lobe: concordant findings from fMRI and memory-impaired patients. Proc. Natl. Acad. Sci. U.S.A. 103, 9351–9356 10.1073/pnas.0602716103 16751272 PMC1482613

[B75] Good I. Johnstrude I. Ashburner J. Henson R. N. A. Friston K. J. Frackowiak R. S. J. (2001). Cerebral asymmetry and the effects of sex and handedness on brain structure: a voxel based morphometric analysis of 465 human brains. Neuroimage 14, 685–700 10.1006/nimg.2001.0857 11506541

[B76] Goodglass H. Kaplan E. F. (1983). Assessment of Aphasia and Related Disorders, 2nd Edn. Philadelphia, PA: Lea and Feibiger

[B77] Goral M. Spiro A. Albert M. L. Oblier L. K. Connor L. (2007). Changes in lexical retrieval ability during adulthood. Ment. Lex. 2, 215–238

[B78] Harati H. Barbelivien A. Cosquer B. Majchrzak M. Cassel J. C. (2008). Selective cholinergic lesions in the rat nucleus basalis magnocellularis with limited damage in the medial septum specifically alter attention performance in the five-choice serial reaction time task. Neuroscience 153, 72–83 18339485 10.1016/j.neuroscience.2008.01.031

[B79] Harati H. Majchrzak M. Cosquer B. Galani R. Kelche C. Cassel J. C. Barbelivien A. (2011). Attention and memory in aged rats: impact of lifelong environmental enrichment. Neurobiol. Aging 32, 718–736 10.1016/j.neurobiolaging.2009.03.012 19398248

[B80] Hasher L. Lustig C. Zacks R. (2007). Inhibitory mechanisms and the control of attention, in Variation in Working Memory, eds Conway A. R. A. Jarrold C. Kane M. J. Miyake A. Towse J. N. (New York, NY: Oxford University Press), 227–249

[B81] Hasher L. Zacks R. T. May C. P. (1999). Inhibitory control, circadian arousal, and age, in Attention and Performance, XVII, Cognitive Regulation of Performance: Interaction of Theory and Application, eds Gopher D. Koriat A. (Cambridge, MA: MIT Press), 653–675

[B81a] Heaton R. K. Miller S. W. Taylor M. J. Grant I. (2004). Revised Comprehensive Norms for an Expanded Halstead-Reitan Battery: Demographically Adjusted neuropsychological Norms for African American and Caucasian Adults. Odessa, FL: Psychological Assessment Resources

[B82] Hedden T. Yoon C. (2006). Individual differences in executive processing predict susceptibility to interference in verbal working memory. J. Neuropsychol. 20, 511–528 10.1037/0894-4105.20.5.511 16938014

[B83] Herrera-Guzmán I. Peña-Casanova J. Lara J. P. Gudayol-Ferré E. Böhm P. (2004). Influence of age, sex, and education on the visual object and space perception battery (VOSP) in a healthy normal elderly population. Clin. Neuropsychol. 18, 385–394 10.1080/1385404049052421 15739810

[B84] Hull R. Martin R. C. Beier M. E. Lane D. Hamilton A. C. (2008). Executive function in older adults: a structural equation modeling approach. J. Neuropsychol. 22, 508–522 10.1037/0894-4105.22.4.508 18590362

[B85] Hutchison K. A. Balota D. A. Duchek J. M. (2010). The utility of stroop task switching as a marker for early-stage Alzheimer's disease. Psychol. Aging 25, 545–559 10.1037/a0018498 20853964 PMC2946107

[B86] Iaria G. Petrides M. Dagher A. Pike B. Bohbot V. D. (2003). Cognitive strategies dependent on the hippocampus and caudate nucleus in human navigation: variability and change with practice. J. Neurosci. 23, 5945–5952 12843299 10.1523/JNEUROSCI.23-13-05945.2003 PMC6741255

[B87] Insel K. Morrow D. Brewer B. Figueredo A. (2006). Executive function, working memory, and medication adherence among older adults. J. Gerontol. Psychol. Sci. Soc. Sci. 61, 102–107 16497953 10.1093/geronb/61.2.p102

[B88] Ivnik R. J. Malec J. F. Smith G. E. Tangalos E. G. Peterson R. C. (1996). Neuropychological test norms above age 55, COWAT, BNT, MAE Token, WRAT-R Reading, AMNART, Stroop, TMT, and JLO. Clin. Neuropsychol. 10, 262–278

[B89] Jack C. R. Jr. Bernstein M. A. Fox N. C. Thompson P. Alexander G. Harvey D. Borowski B. Britson P. J. Whitwell J. Ward C. Dale A. M. Felmlee J. P. Gunter J. L. Hill D. L. Killiany R. Schuff N. Fox-Bosetti S. Lin C. Studholme C. DeCarli C. S. Krueger G. Ward H. A. Metzger G. J. Scott K. T. Mallozzi R. Blezek D. Levy J. Debbins J. P. Fleisher A. S. Albert M. Green R. Bartzokis G. Glover G. Mugler J. Weiner M. W. (2008). The Alzheimer's disease neuroimaging initiative (ADNI): MRI methods. J. Magn. Reson. Imaging 27, 685–691 10.1002/jmri.21049 18302232 PMC2544629

[B90] Jacobs W. J. Laurance H. E. Thomas K. G. F. (1997). Place learning in virtual space I: acquisition, overshadowing, and transfer. Learn. Motiv. 28, 521–541

[B91] Jansen P. Schmelter A. Heil M. (2010). Spatial knowledge acquisition in younger and elderly adults: a study in a virtual environment. Exp. Psychol. 57, 54–60 10.1027/1618-3169/a000007 20178963

[B92] Jennings J. M. Jacoby L. L. (1997). An opposition procedure for detecting age-related deficits in recollection: telling effects of repetition. Psychol. Aging 12, 352–361 9189995 10.1037//0882-7974.12.2.352

[B93] Johnson M. K. Hashtroudi S. Lindsay D. S. (1993). Source monitoring. Psychol. Bull. 114, 3–28 8346328 10.1037/0033-2909.114.1.3

[B94] Jonides J. Smith E. E. Marshuetz C. Koeppe R. A. Reuter-Lorenz P. A. (1998). Inhibition of verbal working memory revealed by brain activation. Proc. Natl. Acad. Sci. U.S.A. 95, 8410–8413 9653200 10.1073/pnas.95.14.8410 PMC20989

[B95] Kail R. (1998). Speed of information processing in patients with multiple sclerosis. J. Clin. Exp. Neuropsychol. 20, 98–106 10.1076/jcen.20.1.98.1483 9672823

[B96] Kail R. Salthouse T. A. (1994). Processing speed as a mental capacity. Acta Psychol. 86, 199–225 7976467 10.1016/0001-6918(94)90003-5

[B97] Kane M. J. Conway A. R. A. Hambrick D. Z. Engle R. W. (2007). Variation in working memory capacity as variation in executive attention and control, in Variation in Working Memory, eds Conway A. R. A. Jarrold C. Kane M. J. Miyake A. Towse J. N. (New York, NY: Oxford University Press), 21–48

[B98] Kaplan E. Goodglass H. Weintraub S. (1983). The Boston Naming Test. Philadelphia, PA: Lea and Febiger

[B99] Kave G. Knafo A. Gilboa A. (2010). The rise and fall of word retrieval across the lifespan. Psychol. Aging 25, 719–724 10.1037/a0018927 20853975

[B100] Kemper S. Thompson M. Marquis J. (2001). Longitudinal change in language production: effects of aging and dementia on grammatical complexisy and propositional content. Psychol. Aging 16, 600–614 11766915 10.1037//0882-7974.16.4.600

[B101] Kemps E. Newsom R. (2006). Comparison of adult age differences in verbal and visuo-spatial memory: the importance of ‘pure’, parallel and validated measures. J. Clin. Exp. Neuropsychol. 28, 341–356 10.1080/13803390490918228 16618624

[B102] Kennedy K. M. Raz N. (2009). Aging white matter and cognition: differential effects of regional variations in diffusion properties on memory, executive functions, and speed. Neuropsychologia 47, 916–927 10.1016/j.neuropsychologia.2009.01.001 19166865 PMC2643310

[B103] Kennedy K. M. Rodrigue K. M. Head D. Gunning-Dixon F. Raz N. (2009). Neuroanatomical and cognitive mediators of age-related differences in perceptual priming and learning. J. Neuropsychol. 23, 475–491 10.1037/a0015377 19586211 PMC2754698

[B104] Kim C. Johnson N. F. Cilles S. E. Gold B. T. (2011). Common and distinct mechanisms of cognitive flexibility in prefrontal cortex. J. Neurosci. 31, 4771–4779 10.1523/JNEUROSCI.5923-10.2011 21451015 PMC3086290

[B105] Kravitz D. J. Saleem K. S. Baker C. I. Mishkin M. I. (2011). A new neuralframework for visuospatial processing. Nat. Rev. Neurosci. 12, 217–230 10.1038/nrn3008 21415848 PMC3388718

[B106] Kyllonen P. C. Christal R. E. (1990). Reasoning ability is (little more than) working memory capacity?! Intelligence 14, 389–433

[B107] LaSarge C. Nicolle M. (2009). Comparison of different cognitive rat models of human aging, in Animal Models of Human Cognitive Aging, eds Bizon J. L. Woods A. G. (New York: Humana Press), 73–102

[B108] Latzman R. D. Markon K. E. (2010). The factor structure and age-related factorial invariance of the Delis-Kaplan executive function system (D-KEFS). Assessment 17, 172–184 10.1177/1073191109356254 20040723

[B109] Lee T. Mosing M. A. Henry J. D. Trollor J. N. Lammel A. Ames D. Martin N. G. Wright M. J. Sachdev P. S. (2012). Genetic influences on five measures of processing speed and their covariation with general cognitive ability in the elderly: the Older Australian Twins Study. Behav. Genet. 42, 96–106 10.1007/s10519-011-9474-1 21617952

[B110] Lezak M. D. Howieson D. B. Bigler E. D. Tranel D. (2012). Neuropsychological Assessment, 5th Edn. New York, NY: Oxford University Press

[B111] Li S. C. Lindenberger U. Sikstrom S. (2001). Aging cognition: from neuromodulation to representation to cognition. Trends Cogn. Sci. 5, 479–486 10.1016/S1364-6613(00)01769-1 11684480

[B112] Libon D. J. Glosser G. Malamute B. L. Barbara L. Kaplan E. Goldberg E. Swenson R. Prouty Sands L. (1994). Age, executive functions, and visuospatial functioning in healthy older adults. J. Neuropsychol. 8, 38–43

[B113] Lyn H. Pierre P. Bennett A. J. Fears S. Woods R. Hopkins W. D. (2011). Planum temporale grey matter asymmetries in chimpanzees (*Pan troglodytes*), vervet (*Chlorocebus aethiops sabaeus*), rhesus (*Macaca mulatta*) and bonnet (*Macaca radiata*) monkeys. Neuropsychologia 49, 2004–2012 10.1016/j.neuropsychologia.2011.03.030 21447349 PMC3151738

[B114] Martin M. Kliegel M. McDaniel M. A. (2003). The involvement of executive function in prospective memory performance of adults. Int. J. Psychol. 38, 195–206

[B115] Mattis S. (1988). Dementia Rating Scale: Professional Manual. Odessa, FL: Psychological Assessment Resources

[B116] May C. P. Hasher L. Kane M. J. (1999). The role of interference in memory span. Mem. Cognit. 27, 759–767 10540805 10.3758/bf03198529

[B117] McDaniel M. A. Glisky E. L. Rubin S. R. Guynn M. J. Routhieaux B. C. (1999). Prospective memory: a neuropsychological study. Neuropsychology 13, 103–110 10067781 10.1037//0894-4105.13.1.103

[B118] McDowd J. Hoffman L. Rozek E. Lyons K. E. Pahwa R. Kemper S. (2011). Understanding verbal fluency in healthy aging, Alzheimer's disease and Parkinson's disease. Neuropsychology 25, 210–225 10.1037/a0021531 21381827

[B119] McFarland C. P. Glisky E. L. (2009). Frontal lobe involvement in a task of time-based prospective memory. Neuropsychologia 47, 1660–1669 10.1016/j.neuropsychologia.2009.02.023 19397861 PMC2691905

[B120] McFarland C. P. Glisky E. L. (2011). Implementation intentions and prospective memory among older adults: an investigation of the role of frontal lobe function. Aging Neuropsychol. Cogn. 18, 633–652 10.1080/13825585.2011.613449 22032198

[B121] Meinzer M. Flaisch T. Wilser L. Eulitz C. Rockstroh B. Conway T. Gonzalez Rothi L. Crosson B. C. (2009). Neural signatures of semantic and phonemic fluency in young and old adults. J. Cogn. Neurosci. 10, 2007–2018 10.1162/jocn.2009.21219 19296728 PMC2730979

[B122] Menich S. R. Baron A. (1984). Social housing of rats: life-span effects on reaction time, exploration, weight, and longevity. Exp. Aging Res. 10, 95–100 10.1080/03610738408258550 6499893

[B123] Meyers J. E. Meyers K. R. (1995). Rey complex figure test and recognition trial: professional manual. PAR, Inc. http://www4.parinc.com

[B124] Miller S. L. Celone K. DePeau K. Diamond E. Dickerson B. C. Rentz D. Pihlajamäki M. Sperling R. A. (2008). Age-related memory impairment associated with loss of parietal deactivation but preserved hippocampal activation. Proc. Natl. Acad. Sci. U.S.A. 105, 2181–2186 10.1073/pnas.0706818105 18238903 PMC2538895

[B125] Mishkin M. Delacour J. (1975). An analysis of short-term visual memory in the monkey. J. Exp. Psychol. Anim. Behav. Process. 1, 326–334 811754 10.1037//0097-7403.1.4.326

[B126] Mitchell K. J. Johnson M. K. Raye C. L. D'Esposito M. (2000). FMRI evidence of age-related hippocampal dysfunction in feature binding in working memory. Cogn. Brain Res. 10, 197–206 10.1016/S0926-6410(00)00029-X 10978709

[B127] Mitchell K. J. Raye C. L. Johnson M. K. Greene E. J. (2006). An fMRI investigation of short-term source memory in young and older adults. Neuroimage 30, 627–633 10.1016/j.neuroimage.2005.09.039 16256377

[B128] Miyake A. Friedman N. P. Emerson M. J. Witzki A. H. Howerter A. (2000). The unity and diversity of executive functions and their contributions to complex “frontal lobe” tasks: a latent variable analysis. Cogn. Psychol. 41, 49–100 10.1006/cogp.1999.0734 10945922

[B129] Moffat S. D. Resnick S. M. (2002). Effects of age on virtual environment place navigation and allocentric cognitive mapping. Behav. Neurosci. 116, 851–859 12369805 10.1037//0735-7044.116.5.851

[B130] Morrell C. H. Gordon-Salant S. Pearson J. D. Brant L. J. Fozard J. L. (1996). Age-and gender-specific reference ranges for hearing level and longitudinal change in hearing level. J. Acoust. Soc. Am. 100, 1949–1967 10.1121/1.417906 8865630

[B131] Morris N. Jones D. M. (1990). Memory updating in working memory: the role of the central executive. Br. J. Psychol. 81, 111–121

[B132] Morris R. G. M. Garrud P. Rawlins J. N. O'Keefe J. (1982). Place navigation impaired in rats with hippocampal lesions. Nature 297, 681–683 7088155 10.1038/297681a0

[B133] Moss M. B. Moore T. L. Schettler S. P. Killiany R. Rosene D. (2007). Successful vs. unsuccessful aging in the rhesus monkey, in Brain Aging: Models, Methods, and Mechanisms, ed Riddle D. R. (Boca Raton, FL: CRC Press), 21–38 21204342

[B134] Moye J. (1997). Nonverbal memory assessment with designs: construct validity and clinical utility. Neuropsychol. Rev. 7, 157–170 9471111 10.1023/b:nerv.0000005907.34499.43

[B135a] Murray E. A. Bussey T. J. (1999). Perceptual-mnemonic functions of the perirhinal cortex. Trends Cogn. Sci. 3, 142–151 10.1016/S1364-6613(99)01303-0 10322468

[B136] Naveh-Benjamin M. (2000). Adult age differences in memory performance: tests of an associative deficit hypothesis. J. Exp. Psychol. Learn. Mem. Cogn. 26, 1170–1187 11009251 10.1037//0278-7393.26.5.1170

[B137] Naveh-Benjamin M. Brav T. K. Levy O. (2007). The associative memory deficit of older adults: the role of strategy utilization. Psychol. Aging 22, 202–208 10.1037/0882-7974.22.1.202 17385995

[B138] Naveh-Benjamin M. Guez J. Kilb A. Reedy S. (2004). The associative deficit of older adults: further support using face-name associations. Psychol. Aging 19, 541–546 10.1037/0882-7974.19.3.541 15383004

[B139] Naveh-Benjamin M. Hussain Z. Guez J. Bar-On M. (2003). Adult age differences in episodic memory: further support for an associative deficit hypothesis. J. Exp. Psychol. Learn. Mem. Cogn. 29, 826–837 10.1037/0278-7393.29.5.826 14516216

[B140] Nedelska Z. Andel R. Lacso J. Vicek K. Horinek D. Lisy J. Shearadova K. Bures J. Hort J. (2012). Spatial navigation impairment is proportional to right hippocampal volume. Proc. Natl. Acad. Sci. U.S.A. 09, 2590–2594. 10.1073/pnas.1121588109 22308496 PMC3289383

[B141] Newman M. C. Kaszniak A. W. (2000). Spatial memory and aging: performance on a human analog of the Morris water maze. Aging Neuropsychol. Cogn. 7, 86–93

[B142] Nielson K. A. Langenecker S. A. Garavan H. (2002). Differences in the functional neuroanatomy of inhibitory control across the adult lifespan. Psychol. Aging 17, 56–71 11931287 10.1037//0882-7974.17.1.56

[B143] Nilsson L. G. Adolfsoon R. Backman L. Cruts M. Edvardsson H. Nyberg L. van Broeckhoven C. (2002). Memory development in adulthood and old age: the Betula prospective cohort study, in Lifespan Development of Human Memor, eds Graf P. Ohta N. (Cambridge, MA: MIT Press), 185–204

[B144] Nyberg L. Maitland S. B. Ronnlund M. Backman L. Dixon R. A. Wahlin A. Nilsson L.-G. (2003). Selective adult age differences in an age-invariant multifactor model of declarative memory. Psychol. Aging 18, 149–160 12641319 10.1037/0882-7974.18.1.149

[B145] Obler L. K. Rykhlevskaia E. Schnyer D. Clark-Cotton M. Spiro A. Hyun J. Kim D. Goral M. Albert M. L. (2010). Bilateral brain regions associated with naming in older adults. Brain Lang. 113, 113–123 10.1016/j.bandl.2010.03.001 20399492 PMC2975055

[B146] Park D. C. Morrel R. W. Frieske D. Kincaid D. (1992). Medication adherence behaviors in older adults: effects of external cognitive supports. Psychol. Aging 7, 252–256 1610514 10.1037//0882-7974.7.2.252

[B147] Park D. C. Reuter-Lorenz P. A. (2009). The adaptive brain: aging and neurocognitive scaffolding. Annu. Rev. Psychol. 60, 173–196 10.1146/annurev.psych.59.103006.093656 19035823 PMC3359129

[B148] Park D. C. Smith A. D. Lautenschalger G. Earles J. Frieske D. Zwahr M. Gaines C. (1996). Mediators of long-term memory performance across the life span. Psychol. Aging 11, 621–637 9000294 10.1037//0882-7974.11.4.621

[B149] Patterson K. Shewell C. (1987). Speak and spell: dissociations and word class effects, in The Cognitive Neuropsychology of Language, eds. Coltheart M. Sartori G. Job R. (London: Erlbaum), 273–294

[B150] Penke L. Maniega S. M. Murray C. Gow A. J. Valdes Hernandez M. C. Clayden J. D. Starr J. M. Wardlaw J. M. Bastin M. E. Deary I. J. (2010). A general factor of brain white matter integrity predicts information processing speed in healthy older people. J. Neurosci. 30, 7569–7574 10.1523/JNEUROSCI.1553-10.2010 20519531 PMC6632368

[B151] Rabbitt P. Lunn M. Pendleton N. Horan M. Scott M. Thacker N. Lowe C. Jackson A. (2007). White matter lesions account for all age-related declines in speed but not intelligence. Neuropsychology 21, 363–370 10.1037/0894-4105.21.3.363 17484599

[B152] Raz N. Ghisletta P. Rodrigue K. M. Kennedy K. M. Lindenberger U. (2010). Trajectories of brain aging in middle-aged and older adults: regional and individual differences. Neuroimage 51, 501–511 10.1016/j.neuroimage.2010.03.020 20298790 PMC2879584

[B153] Raz N. Lindenberger U. Rodrigue K. Kennedy K. M. Head D. Williamson A. Dahle C. Gerstorf D. Acker J. D. (2005). Regional brain changes in aging healthy adults: general trends, individual differences and modifiers. Cereb. Cortex 15, 1676–1689 10.1093/cercor/bhi044 15703252

[B154] Raz N. Rodrigue K. M. (2006). Differential aging of the brain: patterns, cognitive correlates, and modifiers. Neurosci. Biobehav. Rev. 30, 730–748 10.1016/j.neubiorev.2006.07.001 16919333 PMC6601348

[B155] Read D. E. (1988). Age-related changes in performance on a visual-closure task. J. Clin. Exp. Neuropsychol. 10, 451–456 10.1080/01688638808408252 3403707

[B156] Reitan R. (1958). Validity of the trail making test as an indicator of organic brain damage. Percept. Mot. Skills 8, 271–276

[B157] Reitan R. Wolfson D. (1993). The Halstead-Reitan Neuropsychological Test Battery: Theory and Clinical Interpretation. Tucson, AZ: Neuropsychology Press

[B158] Reuter-Lorenz P. Park D. (2010). Human neuroscience and the aging mind: a new look at old problems. J. Gerontol. B. Psychol. Sci. Soc. Sci. 65, 405–415. 10.1093/geronb/gbq035 20478901 PMC2883872

[B159] Robbins T. W. (2002). The 5-choice serial reaction time task: behavioural pharmacology and functional neurochemistry. Psychopharmacology (Berl.) 163, 362–380 10.1007/s00213-002-1154-7 12373437

[B160] Robbins T. W. James M. Owen A. M. Sahakian B. J. McInnes L. Rabbitt P. (1994). Cambridge neuropsychological test automated battery (CANTAB): a factor analytic study of a large sample of normal elderly volunteers. Dementia 5, 266–281 7951684 10.1159/000106735

[B161] Rogers R. D. Monsell S. (1995). Costs of a predictable switch between simple cognitive tasks. J. Exp. Psychol. Gen. 124, 207–231

[B162] Ryan L. Walther K. Bendlin B. B. Lue L.-F. Walker D. G. Glisky E. L. (2011). Age-related differences in white matter integrity measured by diffusion tensor imaging and cognitive function are related to APOE status. Neuroimage 54, 1565–1577 10.1016/j.neuroimage.2010.08.052 20804847 PMC2997188

[B163] Salthouse T. (2009). Decomposing age correlations on neuropsychological and cognitive variables. J. Int. Neuropsychol. Soc. 15, 65–661 10.1017/S1355617709990385 19570312 PMC3633567

[B164] Salthouse T. A. (1993). Speed mediation of adult age differences in cognition. Dev. Psychol. 29, 722–738

[B165] Salthouse T. A. (1996). The processing speed theory of adult age differences in cognition. Psychol. Rev. 103, 403–428 8759042 10.1037/0033-295x.103.3.403

[B166] Salthouse T. A. (2000). Aging and measures of processing speed. Biol. Psychol. 54, 35–54 10.1016/S0301-0511(00)00052-1 11035219

[B168] Salthouse T. A. Meinz E. J. (1995). Aging, inhibition, working memory, and speed. J. Gerontol. B. Psychol. Sci. Soc. Sci. 50, P297–P306 7583809 10.1093/geronb/50b.6.p297

[B170] Schaie K. W. (2005). Developmental Influences on Adult Intelligence: The Seattle Longitudinal Study. New York, NY: Oxford University Press

[B171] Schnabel R. (2012). Overcoming the challenge of re-assessing logical memory. Clin. Neuropsychol. 26, 102–115 10.1080/13854046.2011.640642 22172088

[B172] Schwerdt P. R. Dopkins S. (2001). Memory for content and source in temporal lobe patients. Neuropsychology 15, 48–57 11216889

[B173] Shallice T. Stuss D. T. Alexander M. P. Picton T. W. Derkzen D. (2008). The multiple dimensions of sustained attention. Cortex 44, 794–805 10.1016/j.cortex.2007.04.002 18489960

[B174] Simon J. R. (1990). The effects of an irrelevant directional cue on human information processing, in Stimulus-Response Compatibility: An Integrated Perspective, eds Proctor R. W. Reeves T. G. (Amsterdam: North-Holland), 31–86

[B175] Small S. A. Tsai W. Y. De La Paz R. Mayeux R. Stern Y. (2002). Imaging hippocampal function across the human life span: is memory decline normal or not? Ann. Neurol. 51, 290–295 10.1002/hbm.20349 11891823

[B176] Spencer W. D. Raz N. (1995). Differential effects of aging on memory for content and context: a meta-analysis. Psychol. Aging 10, 527–539 8749580 10.1037//0882-7974.10.4.527

[B177] Sperling R. A. Laviolette P. O'Keefe K. O'Brien J. Rentz D. M. Pihlajamaki M. Marshall G. Hyman B. T. Selkoe D. J. Hedden T. Buckner R. L. Becker J. A. Johnson K. A. (2009). Amyloid deposition is associated with impaired default network function in older persons without dementia. Neuron 63, 178–188 10.1016/j.neuron.2009.07.003 19640477 PMC2738994

[B178] Stamatakis E. A. Shafto M. A. Williams G. Tam P. Tyler L. K. (2011). White matter changes and word finding failures with increasing age. PLos ONE 6:e14496. 10.1371/journal.pone.0014496 21249127 PMC3017545

[B179] Steinmetz H. Fürst G. Meyer B. (1989). Craniocerebral topography within the international 10–20 system. Electroencephalogr. Clin. Neurophysiol. 72, 499–506 2471619 10.1016/0013-4694(89)90227-7

[B180] Stemmer B. Whitaker H. (2008). Handbook of the Neuroscience of Language. London: Academic Press

[B181] Stroop J. R. (1935). Studies of interference in serial verbal reactions. J. Exp. Psychol. 18, 643–662

[B182] Studzinski C. M. Christie L. Araujoa J. A. Burnham J. M. Head E. Cotman C. W. Milgrama N. W. (2006). Visuospatial function in the beagle dog: an early marker of cognitive decline in a model of human aging and dementia. Neurobiol. Learn. Mem. 86, 197–204 10.1016/j.nlm.2006.02.005 16616528

[B183] Stuss D. T. (2011). Functions of the frontal lobes: relation to executive functions. J. Int. Neuropsychol. Soc. 17, 759–765 10.1017/S1355617711000695 21729406

[B184] Stuss D. T. Alexander M. P. (2007). Is there a dysexecutive syndrome? Philos. Trans. R. Soc. Lond., B, Biol. Sci. 362, 901–915 10.1098/rstb.2007.2096 17412679 PMC2430005

[B185] Stuss D. T. Levine B. (2002). Adult clinical neuropsychology: lessons from studies of the frontal lobes. Annu. Rev. Psychol. 53, 401–433 10.1146/annurev.psych.53.100901.135220 11752491

[B186] Sylvester C.-Y. C. Wager T. D. Lacey S. C. Hernandez L. Nichols T. E. Smith E. E. Jonides J. (2003). Switching attention and resolving interference: fMRI measures of executive functions. Neuropsychologia 41, 357–370 10.1016/S0028-3932(02)00167-7 12457760

[B187] Takeuchi H. Taki Y. Hashizume H. Sassa Y. Nagase T. Nouchi R. Kawashima R. (2011). Effects of training of processing speed on neural systems. J. Neurosci. 31, 12139–12148 10.1523/JNEUROSCI.2948-11.2011 21865456 PMC6623215

[B188] Troyer A. K. Moscovitch M. Winocur G. (1997). Clustering and switching as two components of verbal fluency: evidence from younger and older healthy adults. Neuropsychology 11, 138–146 9055277 10.1037//0894-4105.11.1.138

[B189] Tulving E. (1972). Episodic and semantic memory, in Organization of Memory, eds Tulving E. Donaldson W. (New York, NY: Academic Press), 381–403

[B190] Tulving E. (2002). Episodic memory: from mind to brain. Annu. Rev. Psychol. 53, 1–25 10.1146/annurev.psych.53.100901.135114 11752477

[B191] Turner M. L. Engle R. W. (1989). Is working memory capacity task dependent? J. Mem. Lang. 28, 127–154

[B192] Vaughan L. Giovanello K. (2010). Executive function in daily life: age-related influences of executive processes on instrumental activities of daily living. Psychol. Aging 25, 343–355 10.1037/a0017729 20545419

[B193] Verhaeghen P. (2003). Aging and vocabulary score: a meta-analysis. Psychol. Aging 16, 371–384 12825780 10.1037/0882-7974.18.2.332

[B194] Verhaeghen P. Cerella J. (2002). Aging, executive control, and attention: a review of meta-analyses. Neurosci. Biobehav. Rev. 26, 849–857 10.1016/S0149-7634(02)00071-4 12470697

[B195] Wagster M. V. (2009). Cognitive aging research: an exciting time for a maturing field: a postscript to the special issue of neuropsychology review. Neuropsychol. Rev. 19, 523–525 10.1007/s11065-009-9121-2 19943133

[B196] Wechsler D. (1987). Wechsler Memory Scale—Revised. New York, NY: Psychological Corporation

[B197] Wechsler D. (1997). Wechsler Adult Intelligence Scale—III. San Antonio, TX: Psychological Corporation, Harcourt Brace

[B198] Wechsler D. (2008). Administration and Scoring Manual for the Wechsler Adult Intelligence Scale – IV. San Antonio, TX: NCS Pearson, Inc

[B199] Weirenga C. E. Benjamin M. Gopinath K. Perlstein W. Leonard C. M. Gonzalez Rothi L. J. Conway T. Cato M. A. Briggs R. Crosson B. (2008). Age-related changes in word retrieval: role of bilateral frontal and subcortical networks. Neurobiol. Aging 29, 436–451 10.1016/j.neurobiolaging.2006.10.024 17147975

[B200] Welch L. W. Doineau D. Johnson S. King D. (1996). Eudcational and gender normative data from the Boston naming test in a group of older adults. Brain Lang. 53, 260–266 10.1006/brln.1996.0047 8726536

[B201] Wiig E. H. Nielsen N. P. Jacobson J. M. (2007). A quick test of cognitive speed: patterns of age groups 15 to 95 years. Percept. Mot. Skills 104, 1067–1075 17879639 10.2466/pms.104.4.1067-1075

[B202] Wingfield A. (1996). Cognitive factors in auditory performance: context, speed of processing, and constraints of memory. J. Am. Acad. Audiol. 7, 175–182 8780990

[B203] Wingfield A. Grossman M. (2006). Language and the aging brain: patterns of neural compensation revealed by functional brain imaging. Neurophysiology 96, 2830–2839 10.1152/jn.00628.2006 17110737

[B204] Wingfield A. Tun P. A. Koh C. K. Rosen M. J. (1999). Regarding lost time: adult aging and the effect of time restoration on recall of time-compressed speech. Psychol. Aging 14, 380–389 10509694 10.1037//0882-7974.14.3.380

[B205] Winstanley C. A. Eagle D. M. Robbins T. W. (2006). Behavioral models of impulsivity in relation to impulsivity in ADHD: translation between clinical and preclinical studies. Clin. Psychol. Rev. 26, 379–395 10.1016/j.cpr.2006.01.001 16504359 PMC1892795

[B206] Zec R. F. (1995). The neuropsychology of aging. Exp. Gerontol. 30, 431–442 7556520 10.1016/0531-5565(94)00066-c

[B207] Zec R. F. Markwell S. J. Burkett N. R. Larsen D. L. (2005). A longitudinal study of confrontation naming in the normal elderly. J. Int. Neuropsychol. Soc. 11, 716–726 10.1017/S1355617705050897 16248907

